# Prelamin A processing, accumulation and distribution in normal cells and laminopathy disorders

**DOI:** 10.1080/19491034.2016.1150397

**Published:** 2016-02-22

**Authors:** Andrea Casasola, David Scalzo, Vivek Nandakumar, Jessica Halow, Félix Recillas-Targa, Mark Groudine, Héctor Rincón-Arano

**Affiliations:** a Basic Science Division, Fred Hutchinson Cancer Research Center, Seattle, WA, USA; b Department of Radiation Oncology, University Washington School of Medicine, Seattle, WA, USA; c Instituto Fisiología Celular, Universidad Nacional Autónoma de México, Mexico City, Mexico

**Keywords:** intracellular flow cytometry (IFC), lamin A, monoclonal antibody, nuclear envelope, prelamin A, post-translational processing, progeriod syndromes, ZMPSTE24

## Abstract

Lamin A is part of a complex structural meshwork located beneath the nuclear envelope and is involved in both structural support and the regulation of gene expression. Lamin A is initially expressed as prelamin A, which contains an extended carboxyl terminus that undergoes a series of post-translational modifications and subsequent cleavage by the endopeptidase ZMPSTE24 to generate lamin A. To facilitate investigations of the role of this cleavage in normal and disease states, we developed a monoclonal antibody (PL-1C7) that specifically recognizes prelamin A at the intact ZMPSTE24 cleavage site, ensuring prelamin A detection exclusively. Importantly, PL-1C7 can be used to determine prelamin A localization and accumulation in cells where lamin A is highly expressed without the use of exogenous fusion proteins. Our results show that unlike mature lamin A, prelamin A accumulates as discrete and localized foci at the nuclear periphery. Furthermore, whereas treatment with farnesylation inhibitors of cells overexpressing a GFP-prelamin A fusion protein results in the formation of large nucleoplasmic clumps, these aggregates are not observed upon similar treatment of cells expressing endogenous prelamin A or in cells lacking ZMPSTE24 expression and/or activity. Finally, we show that specific laminopathy-associated mutations exhibit both positive and negative effects on prelamin A accumulation, indicating that these mutations affect prelamin A processing efficiency in different manners.

## Introduction

The nuclear lamina, which lies beneath the inner nuclear membrane, is a relatively insoluble fibrous structure resistant to extraction by detergents.^1^ This lamina is a complex protein meshwork composed of class V intermediate filaments called lamins. Lamins play diverse roles in nuclear organization and in various essential cellular functions such as DNA replication, transcription, and RNA processing.^2-5^ Lamins are divided into 2 subgroups: A- and B-type lamins. In mammals, A-type Lamins (lamins A, C, AΔ10 and C2) are generated by alternative splicing of a single *LMNA* gene. B-type lamins, on the other hand, are encoded by the *LMNB1* and *LMNB2* genes.^6-8^ In general, lamins exhibit 3 distinct structural domains: 1) a conserved central rod domain consisting of 4 helical coils (1A, 1B, 2A, 2B), 2) a conserved immunoglobulin-like domain and 3) a conserved CAAX motif in the carboxyl-terminal end that is isoprenylated and crucial for the attachment to the nuclear envelope, except for lamin C, which lacks the CAAX motif.^9-13^

**Figure 1.**
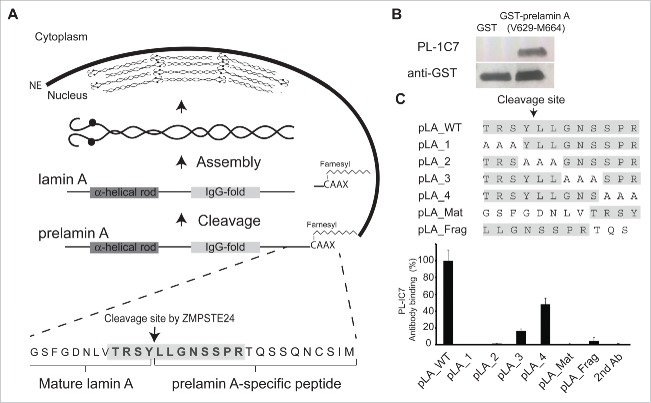
PL-1C7 monoclonal antibody specifically recognizes the lamin A precursor, prelamin A. (A). Diagram of prelamin A structure and processing. The PL-1C7 monoclonal antibody was raised using as an antigen a synthetic peptide composed of the 12 amino acids spanning the ZMPSTE24 cleavage site in prelamin A (T643-R654), and includes 4 amino acids from the mature lamin A as well as 8 specific prelamin A residues (gray shading). (B). PL-1C7 antibody binding to the carboxyl terminus of prelamin A was confirmed by western blot using a GST_prelamin A V629-M664 fusion protein, right lane; non-fusion protein GST control, left lane. (C). PL-1C7 epitope mapping was done by ELISA immunoassays using a panel of 7 synthetic peptides where wild type amino acids triplets were sequentially replaced by alanine triplets, as well as 2 peptide mimics of ZMPSTE24-generated lamin A fragments: pLA_Mat (G635-Y646) and pLA_frag (L647-S657). Antibody binding was plotted as percentage of binding in relation to the wild type lamin A peptide (pLA_WT). p < 0.005. See also Fig. S1.

Lamin A has been intensely studied during the past 4 decades, as it plays a key role in a wide variety of cellular processes, including telomere maintenance, nuclear compartmentalization and DNA repair.^3-5^ Mutations in this structural protein are associated with rare diseases known as laminopathies and in some cases have been proven to affect lamin A post-translational processing. Lamin A is initially synthesized as a precursor known as prelamin A, which undergoes a complex set of modifications in the carboxyl terminus. These modifications, which are required for incorporation of prelamin A into the nuclear envelope and its subsequent processing into the mature lamin A include: 1) addition of a farnesyl isoprenoid group to C662 of the CAAX motif; 2) endoproteolysis of the last 3 amino acids, S663, I664 and M665, resulting in a 662-residue peptide; 3) carboxymethylation of the last residue (C662) by the isoprenylcysteine carboxyl methyltransferase enzyme, and, lastly, 4) cleavage of the 15 C-terminal residues (Y647-C662) by ZMPSTE24, which generates a final protein product containing 646 residues^14-16^ (Fig. 1A). While the first 3 modifications render the carboxyl terminus more hydrophobic, facilitating interactions with the nuclear membrane,^17^ the proteolytic cleavage by ZMPSTE24 produces mature lamin A, which is then incorporated into the nuclear lamina.^9,15,18,19^ However, lamin C is not farnesylated and yet is still located at the nuclear periphery, therefore the role of the farnesyl anchor in nuclear envelope targeting of the lamins remains unclear.^20^

To date, there are over 460 known mutations in the human *LMNA* gene that are associated with laminopathies (The UMD-lamin A mutations database http://www.umd.be/LMNA/). These include Emery-Dreifuss muscular dystrophy, Limb-girdle muscular dystrophy and Mandibuloacral dysplasia, as well as the premature aging disorder Hutchinson-Gilford Progeria Syndrome (HGPS).^21-25^ Importantly, current data demonstrate that HGPS phenotypes are the result of alterations in the prelamin A processing pathway. For example, the most common HGPS mutation, G608G, activates a cryptic splice site in exon 11 of the *LMNA* gene (LMNAΔ50). This leads to the synthesis of an alternatively spliced lamin A variant (progerin) that lacks the recognition site for ZMPSTE24.^25^ While Progerin can be farnesylated, it cannot be cleaved and remains permanently attached to the nuclear envelope.^25-27^ Progeriod nuclei exhibit morphological defects such as changes in size and shape, as well as alterations in cell division and proliferation rates,^27-30^ suggesting that the abnormal accumulation of the farnesylated prelamin A affects cell homeostasis. Using GFP protein fusions, it has been shown that farnesylation inhibitors (FTinh) can block prelamin A association with the nuclear envelope causing the nucleoplasmic aggregation of prelamin A but rescuing the misshapen nuclear phenotype in HGPS cells.^26,31-34^ Similar precursor accumulation was observed with untagged prelamin A upon FTinh treatment, but without the formation of aggregates, suggesting that prenylation is required for targeting prelamin A to the nuclear envelope.^35,36^ Currently, farnesylation inhibitors are being used as a treatment option in HGPS patients.^37-39^ Nevertheless, another study found that the expression of a non-farnesylated prelamin A in *Lmna*^*nPLAO*^ mice did not exhibit progeria-associated phenotypes, but instead caused cardiomyopathy. Importantly, using a rat monoclonal antibody generated against the last 15 residues of prelamin A (clone 7G11)^40^ Davies *et al.* showed that this non-farnesylated prelamin A version can still localize at the nuclear rim.^41^ Similar results were obtained upon knockout of both farnesyl transferase and generalyl transferase proteins (the 2 major prenylating proteins) in keratinocytes resulting in prelamin A accumulation, in particular at the nuclear rim.^40,41^ Together, these genetic results support the notion that addition of the farnesylation anchor is not the determinant step in prelamin A accumulation at the nuclear periphery, but nevertheless, its accumulation at the periphery, whether farnesylated or not, can be toxic.

Although LMNAΔ50-based HGPS is one of the most studied laminopathies, several missense point mutations along the *LMNA* gene have also been found to be associated with the development of Progeriod phenotypes. Collectively, these are known as Atypical Progeriod Syndromes (APS) and are also characterized by nuclear shape abnormalities and cellular toxicity.^42-46^ Phenotypes of APS are more tissue-restricted, observed in particular in skeletal muscle, cardiac muscle, epithelial and vascular tissue.^43,46-49^ In contrast to HGPS, however, APS *LMNA* mutations are not exclusively localized in the carboxyl-terminal end of the protein and the mechanisms by which these mutations lead to their associated defects remains unclear. Additionally, FTinh treatment has not proven to be a successful therapy for APS.^43^

While several studies have revealed morphological differences in nuclei due to the expression of GFP-tagged prelamin A, endogenous prelamin A detection has not been addressed in a quantitative manner. However, the latter has been difficult to measure due to the low prelamin A concentration, as well as its transient state, its low tissue specific expression and the minimal difference in mass with the mature molecule. Here, we report the development and characterization of the monoclonal antibody PL-1C7, which specifically recognizes prelamin A at the region targeted by ZMPSTE24, thus detecting all prelamin A intermediates but not the mature form. We show that PL-1C7 can be effectively used to monitor prelamin A levels by intracellular flow cytometry (IFC) and can also potentially be used as a sensor of ZMPSTE24 activity. We find that prelamin A is incorporated into localized regions of the nuclear periphery in murine cells, and that inhibition of prelamin A farnesylation results in a diffuse distribution of prelamin A within the intranuclear space but not in the formation of prelamin A aggregates (which do form when overexpressing GFP-prelamin A fusion proteins) in both murine and human cells. Interestingly, analysis of laminopathy-associated *LMNA* mutations with the PL-1C7 antibody suggests that while specific mutants cause prelamin A accumulation, others make prelamin A processing more efficient when expressed in lamin A null cells. These results also demonstrate that the PL-1C7 antibody is a useful tool to study prelamin A biology.

## Results

### The monoclonal PL-1C7 antibody recognizes prelamin A at the ZMPSTE24 cleavage site

Several polyclonal anti-lamin A antibodies are commercially available, but all target at least some epitopes within the mature lamin A protein. In contrast, a rat prelamin A monoclonal antibody (7G11) distinguishes between the precursor and mature lamin A forms (Fig. 1A). However, 7G11 also detects the fragment released after ZMPSTE24 cleavage from prelamin A. Thus we were interested in producing an antibody that recognizes only the full length prelamin A. Using the synthetic peptide TRSYLLGNSSPR, which corresponds to the conserved carboxyl terminal region of prelamin A (T643-R654 residues) that spans the ZMPSTE24 cleavage site (Fig. 1A, S1A), we produced the mouse monoclonal IgG2bκ antibody PL-1C7 against prelamin A (Fig. S1B). The specificity of this antibody for the lamin A carboxyl domain was confirmed by the recognition of a GST-prelamin A V629-M664 fusion protein (Fig. 1B).

Since the antigenic sequence used to develop the PL-1C7 antibody also possesses 4 amino acids that remain in the mature product (**TRSY**LLGNSSPRSQSSQNCSIM), we sought to identify the minimal region of recognition by PL-1C7. To accomplish this, we performed epitope mapping analyses evaluating PL-1C7 binding to a set of 5 synthetic peptides: a prelamin A peptide covering the first 12 amino acids of the original antigenic sequence (T643-R654, pLA_WT) and 4 peptides with sequential triplet alanine substitutions along the twelve-amino acid sequence (pLA_1, pLA_2, pLA_3, pLA_4) (Fig. 1C). These experiments reveal that, while PL-1C7 binds to the wild type prelamin A peptide, alanine substitutions in peptides pLA_1 and pLA_2 abolished PL-1C7 binding (Fig. 1C). In addition, when alanine triplets were substituted at sites downstream of the ZMPSTE24 cleavage site, in the pLA_3 or pLA_4 peptide, recognition by PL-1C7 was disrupted 80% and 50%, respectively. These results reveal that the primary PL-1C7 epitope overlaps with the 5 amino acids located at the core of ZMPSTE24 cleavage site (TRSYLL). To determine if PL-1C7 recognizes the 2 fragments generated by this endopeptidase, we tested 2 peptides corresponding to the sequences flanking the ZMPSTE24 cleavage site. One of these peptides represents the carboxyl terminus of the mature lamin A product (pLA_Mat, G635-Y646) and the other represents the fragment released after cleavage by ZMPSTE24 (pLA_frag, L647-S657) (Fig. 1A, C). Our results indicate that PL-1C7 does not recognize ZMPSTE24 generated fragments, as binding to pLA_Mat and pLA_Frag peptides was reduced by 100% and ˜95%, respectively, as compared to binding to the complete original antigenic sequence. Consistent with its preferential binding to the intact ZMPSTE24 cleavage site (TRSYLL), pLA_Mat and pLA_Frag peptides did not compete for PL-1C7 binding to the intact sequence in competition binding assays (Fig. S1C). Importantly, increased PL-1C7 concentrations did not impact its specificity for the epitope, suggesting high specificity for its target sequence (Fig. S1D). In contrast, the previously reported rat prelamin A antibody, 7G11, binds only the fragment released after cleavage by ZMPSTE24 close to the farnesylation sequence (Fig. S1E-F). As the 7G11 antibody recognizes both human and mouse, this antibody must target the Q657S658 sequence, as T656 is not conserved in the mouse epitope used to produce this antibody (Fig. S1A). In summary these findings reveal that the PL-1C7 antibody selectively identifies the carboxyl terminus of prelamin A by recognizing the sequence TRSYLL and, unlike the 7G11 antibody, PL-1C7 does not detect any of the lamin A processing fragments generated from the cleavage by ZMPSTE24.

### Quantitative detection of prelamin A using the PL-1C7 antibody

**Figure 2.**
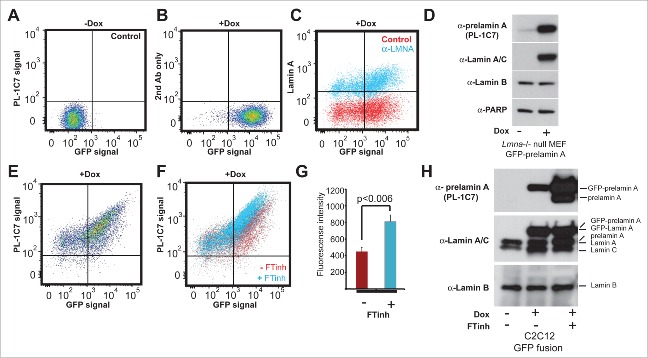
Quantitative detection of prelamin A by intracellular flow cytometry (IFC). Prelamin A is detected by PL-1C7 antibody in *Lmna*^−/−^ MEFs stably transfected with Doxycycline (Dox)-inducible *GFP-Lmna* transgene using IFC. (A). Control, non-induced *GFP-Lmna* MEFs (“x” axis, GFP-fusion; “y” axis, prelamin A signal detected using PL-1C7 antibody). (B). Control *GFP-Lmna* MEFs after 24 hr Dox treatment stained with secondary antibody only (No PL-1C7). (C). Detection of both precursor and processed *Lmna* gene products (lamin A) with anti-lamin A/C antibody in *GFP-Lmna* MEFs after 24 hr Dox treatment. (D). Western blot analysis of *GFP-Lmna* MEF cells treated with Dox for 24h. GFP signal is present on mature lamin A as well as prelamin A. Prelamin A accumulation was detected using the PL-1C7 antibody. Antibodies against lamin A/C, lamin B and PARP1 were used as controls. (E). Dox-treated *GFP-Lmna* MEF stained with PL-1C7 antibody (prelamin A). (F). Farnesyl transferase inhibitor (FTinh) induced prelamin A accumulation in *GFP-Lmna* MEFs detected by IFC using the PL-1C7 antibody (G). Fluorescence geometric median of prelamin A detection using PL-1C7 by IFC after FTihn treatment of *GFP-Lmna* MEFs. (H). Western blot analysis to detect prelamin A accumulation in Dox induced *GFP-Lmna* C2C12 myoblasts upon FTinh treatment. Antibodies against lamin A/C and lamin B were used as controls. See also Fig. S2.

To further examine PL-1C7 specificity and to determine whether this antibody can be used for quantitative detection of prelamin A levels in cells, we performed intracellular flow cytometry (IFC) in *Lmna*^−/−^ mouse embryonic fibroblasts (MEF) stably transfected with a Doxycycline (Dox)-inducible GFP-*Lmna* transgene (*GFP-Lmna* MEF).^50^ After 24 h in Dox, GFP-lamin A can be detected by flow cytometry, both by GFP fluorescence (Fig. 2A-B, S2A) or by using commercial antibodies to lamin A/C (99.8% positive cells) and confirmed by western blot (Fig. 2C-D). IFC validation was also accomplished by staining non-Dox treated *GFP-Lmna* MEFs with an antibody against lamin B, which is constitutively expressed in these cells (Fig. S2B-D). Importantly, staining of Dox-treated cells with PL-1C7 revealed that prelamin A was present in >90% of GFP positive cells, suggesting that both the precursor and lamin A are actively produced in these cells (Fig. 2C-E, S2A). As farnesyltransferase inhibitors (FTinh) can increase prelamin A accumulation, we treated *GFP-Lmna* MEFs with Dox and the FTinh, Lonafarnib, and then determined whether the PL-1C7 antibody could detect variations in prelamin A abundance. IFC showed that, upon FTinh treatment, PL-1C7 detected a 2-fold increase in fluorescence intensity (Geometric mean: 430 (Control) vs 789 (FTinh)) (Fig. 2F-G, S2E). The percentage of positive cells remained unaffected, indicating that PL-1C7 detected the FTinh-induced prelamin A accumulation previously reported by others.^26,31,33^ To validate and extend these results, protein gel blots were performed with nuclear proteins from C2C12 cells transfected with the same *GFP-Lmna* fusion-encoding plasmids used in MEFs. After treating control and transfected cells with Dox and FTinhs, similar increases in GFP-prelamin A and prelamin A were observed (Fig. 2H). Together, these results show that PL-1C7 antibody is a useful tool to detect and measure prelamin A.

### Localized distribution of prelamin A along the nuclear envelope

**Figure 3.**
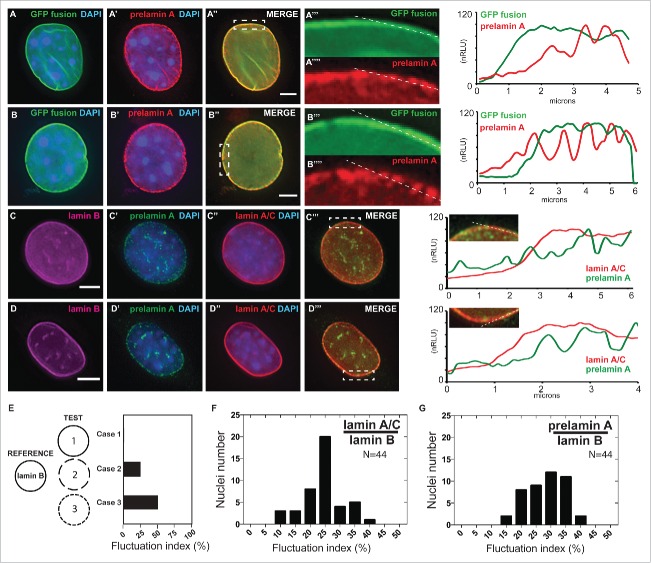
Localized prelamin A detection around the nuclear periphery via immunohistochemical analysis using the PL-1C7 antibody. (A) and (B). Two examples of Dox-treated *GFP-Lmna* MEF cells stained with prelamin A antibody PL-1C7 (red) and counter-stained with DAPI. GFP signal represents preferentially the mature lamin A, but also the prelamin A fraction due to the GFP tag in the N-terminus (Fig. 2). Dotted boxes show regions where correlation analyses between GFP-lamin A and GFP-prelamin A were performed (dotted line). Signal intensities were normalized to the highest value (100). The signal distribution pattern of the GFP-fusion proteins (GFP signal) representing primarily mature lamin A/C (green line; GFP fusion) is significantly different from the prelamin A distribution pattern detected by the PL-1C7 antibody (red line; prelamin A). (C) and (D). Immunostaining for lamin B, lamin A/C and prelamin A (PL-1C7) in wild-type C2C12 cells. Lamin A/C and prelamin A distribution around the nuclear periphery was analyzed as described in (A) and (B). Scale bar: 5 μm. (E). Simulated measurements to show the utility of the ‘fluctuation index’ metric to assess differences in spatial localization of a target protein relative to a reference protein. The fluctuation index increases as the TEST localization pattern becomes increasingly punctate (Case 1, 2 and 3) relative to a reference pattern. (F). Lamin A/C /lamin B fluctuation index in immunostained C2C12 nucleus (n = 44). (G). Prelamin A/lamin B fluctuation index in immunostained C2C12 nucleus (n = 44).

Several studies have demonstrated the continuous distribution of lamin A at the nuclear periphery under normal conditions,^51,52^ but it is unclear whether prelamin A is also homogenously incorporated around the nuclear periphery. To determine prelamin A distribution at the nuclear envelope, we performed indirect immunofluorescence staining using the PL-1C7 antibody in Dox-induced *GFP-Lmna* MEFs. Interestingly, while GFP-lamin A localizes homogenously along the nuclear periphery, the GFP-prelamin A fraction is detected as a well-localized punctate pattern at the periphery, suggesting a more localized incorporation of the lamin A precursor (Fig. 3A-B). To exclude the possibility of this observation arising from the overexpression of *GFP-Lmna* transgene, we performed similar analyses in the myoblast cell line C2C12, as muscle cells tend to be under higher physical stress and exhibit higher lamin A levels.^53^ Consistently, the endogenous lamin A-type proteins (lamin A/C and prelamin A) were detected in the nuclear periphery in these cells. However, prelamin A staining again revealed a more punctate localization pattern relative to lamin A, which is distributed homogenously around the nuclear periphery (Fig. 3C-D). To validate our observation, we developed a quantitative image analysis that generates a fluctuation index metric of endogenous lamin A/C and prelamin A signals around the nuclear envelope. Our custom/novel ‘fluctuation index’ metric compared the localization patterns of lamin A/C or prelamin A by measuring the extent of their signal fluctuation relative to lamin B at the nuclear envelope in microscopy images (see Materials and Methods). For example, a protein (either lamin A or prelamin A) that has a spatial distribution around the nuclear envelope identical to a reference protein (such as lamin B; LMNB) will have a fluctuation index of 0 (Fig. 3E, Case 1). In contrast, when the relative overlap is less, the fluctuation index will increase accordingly (Fig. 3E, Case 2, 3). Our analyses confirm that prelamin A staining exhibits higher fluctuation index values than lamin A/C staining in C2C12 cells, suggesting a more punctate distribution for prelamin A than its mature form (Fig. 3F-G). Together, these results support the idea that, upon synthesis and nuclear translocation, prelamin A is recruited to specific areas of the nuclear periphery to promote its processing and assembly into the nuclear lamina.

### Farnesylation inhibition promotes endogenous prelamin A diffusion over the nucleoplasm but not large aggregates

**Figure 4.**
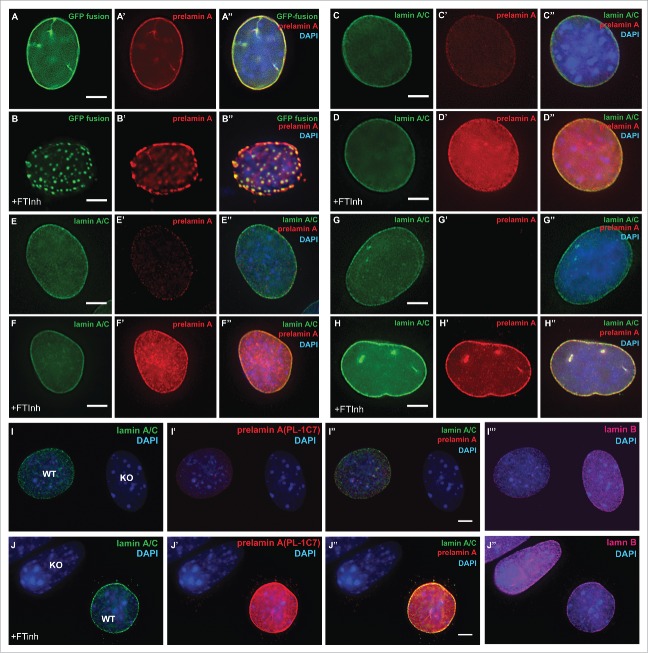
Farnesylation inhibition of endogenous prelamin A causes prelamin A accumulation but not large nucleoplasmic aggregates are not observed. (A) and (B). Dox-induced *GFP-Lmna* MEFS with and without FTinh (Lonafarnib) treatment were stained with PL-1C7 antibody. (C) and (D). Wild-type MEFs plus and minus FTinh treatment were stained with anti-lamin A/C (green) and anti-prelamin A PL-1C7 (red) antibodies. (E) and (F). Prelamin A and lamin A/C detection in C2C12 myoblasts with and without FTinh treatment stained as in D. (G) and (H). Rhabdomyosarcoma (A-204) cells processed as in E and F. (I) and (J). Co-cultured *Lmna*^−/−^ (KO) and wild type (WT) MEFs with or without FTinh treatment stained with PL-1C7, anti-lamin A/C and anti-lamin B antibodies. Anti-lamin A/C antibody was used to distinguish KO from WT cells. See also Fig. S3.

Farnesyltransferase Inhibitors (FTinh) block prelamin A isoprenylation causing its accumulation in the nucleoplasm and, based on analysis of GFP-fusion constructs, the formation of large clumps.^31,33,54^ To determine whether prelamin A is the major component in these clumps, we treated *GFP-Lmna* MEFs with Dox and Lonafarnib for 24 h. GFP-prelamin A staining with the PL-1C7 antibody confirmed that Lonafarnib causes the formation of large aggregates of GFP-prelamin A (Fig. 4A-B). To determine whether this phenotype is observed with the endogenous prelamin A, wild type MEFs and myoblast C2C12 cells were treated with Lonafarnib for 24 h and co-stained with PL-1C7 antibody and lamin A/C specific antibodies. While FTinh caused a global increase in the amount of prelamin A as previously reported (Fig. 2C-F, S3A),^26,33^ our results revealed that the FTinh treatment result in a global diffusion of prelamin A throughout the nucleus but not the formation of large aggregates observed with the GFP-prelamin A fusion proteins (Fig. 4C-F).^26^ These results are consistent with results from the Sinensky lab,^35,36^ but by using our anti-prelamin A specific antibody, we found that FTinh does not seem to collapse or rearrange the prelamin A or lamin A already localized at the periphery, as it is possible to detect its presence at the nuclear rim. To determine whether the observed FTinh-dependent prelamin A diffuse distribution is independent of a fixation protocol, we also performed an immunodetection of prelamin A in C2C12 cells fixed with methanol and observed no differences between the 2 staining protocols (Fig. S3B-E). Importantly, similar results were obtained upon FTinh treatment of several human cell lines from different transformed tissues including Rhabdomyosarcoma (A-204), bone osteosarcoma (U-2 OS), cervix adenocarcinoma (HeLa) and human foreskin fibroblasts (HFF) (Fig. 4G-H, S3A, F-K). Our findings suggest that inhibition of farnesylation causes a diffuse nucleoplasmic accumulation of endogenous prelamin A, but not its aggregation. The observed FTinh-dependent prelamin A aggregation in *GFP-Lmna* expressing MEFs may be a consequence of 2 factors: 1) the GFP tag enhances/promotes prelamin A aggregation^26,31,33^ and/or 2) the higher level expression of the transfected lamin A/prelamin A compared to the low level expression of the endogenous protein.

In addition, while we could detect prelamin A in MEF and C2C12 cells without FTinh treatment, low or no prelamin A detection was observed in the human cells tested (Fig. 4C-H, S3A-F). To make certain that the staining observed in mouse cells is specific for prelamin A, we co-cultured wildtype and *Lmna*^−/−^ MEF with and without FTinh. These cells were stained with an anti-Lamin A/C antibody to distinguish the 2 genetic backgrounds and co-stained with anti-prelamin A PL-1C7 antibody. Our results show that PL-1C7 detects prelamin A in wildtype cells but not in knockout cells, and that FTinh treatment increases PL-1C7 signal only in wildtype cells (Fig. 4I-J). Additional experiments were performed in parallel with the anti-prelamin A antibody 7G11, but detection of prelamin A was observed only upon FTinh treatment as previously reported (Fig. S3L-M).^40^ Interestingly, similar results were obtained by western blot, as we can detect low levels of prelamin A from total C2C12 extracts with the PL-1C7 but not with the rat monoclonal antibody 7G11(Fig. S3L). These results suggest that the PL-1C7 antibody has higher sensitivity in detecting the transient low levels of prelamin A.

### PL-1C7 antibody identifies different effects on prelamin A accumulation based on the type of mutation

**Figure 5.**
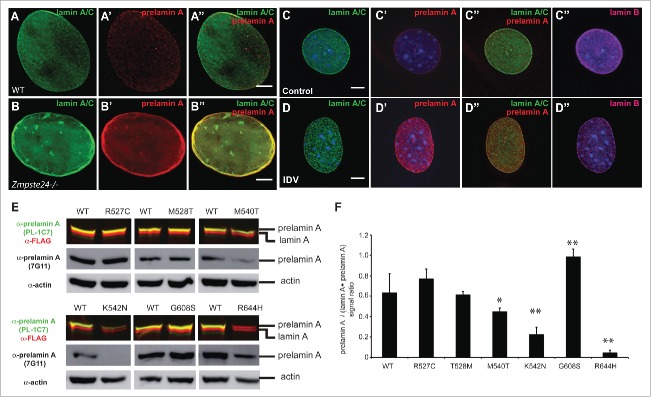
Lack of ZMPSTE24 expression or activity increases prelamin A levels, but laminopathy -associated missense lamin A mutations exert different effects on prelamin A accumulation. (A) and (B). *Zmpste24*^−/−^ and wild type MEFs were co-stained with anti-lamin A/C (green) and anti-prelamin A (Red) antibodies. Increased prelamin A levels can be observed in the absence of the sequence specific protease. (C) and (D). C2C12 cells were treated with the HIV protease inhibitor indinavir (IDV), which inhibits ZMPSTE24 activity. Cells were co-stained as described in A and including an anti-lamin B antibody as control. (E). Analysis of prelamin A accumulation in laminopathy-associated missense lamin A mutations. Dual infrared immunoblots of total proteins from cells transfected with 3XFLAG-tagged human *LMNA* constructs containing different laminopathy-associated mutations including: R527C, T528M, M540T, K542N, G608S and R644H. Blot shows the anti-prelamin A PL-1C7 antibody in green (800 nm channel) and a rabbit anti-FLAG antibody in red (700 nm channel). Membranes were re-blotted with anti-prelamin A 7G11 and β-actin antibodies (loading control) and evaluated by chemiluminescence. (F). Quantification of prelamin A levels in laminopathy-associated mutations. Ratio of prelamin A (800 nm channel)/ Total lamin A/prelamin A (700 nm channel) is shown. Values represent the mean +/− SD, * p< 0.005, ** p< 0.001. See also Fig. S4.

Progeriod syndromes are complex genetic/metabolic disorders that can directly or indirectly affect lamins. For example, restrictive dermopathy, a more severe progeroid syndrome, is caused by a deficiency in ZMPSTE24, which impairs lamin A maturation resulting in an accumulation of a farnesylated and methylated prelamin A.^18,55-57^ To determine whether PL-1C7 can be used as a sensor of ZMPSTE24 activity, we co-stained *Zmpste24*^−/−^ MEFs with anti-prelamin A antibody PL-1C7 and anti-lamin A/C antibody. Western blotting revealed increased levels of prelamin A, and immunostaining experiments showed extensive prelamin A accumulation at the nuclear periphery in these fibroblasts (Fig. 5A-B, S4A). To confirm that PL-1C7 is an efficient tool to detect ZMPSTE24 activity, we treated C2C12 cells with the HIV protease inhibitor Indinavir, which blocks ZMPSTE24,^58,59^ and immunostained with PL-1C7. Consistent with our previous results, indinavir caused the accumulation of prelamin A in myoblast cells (Fig. 5C-D). These microscopy results along with PL-1C7 epitope characterization demonstrate that PL-1C7 can be used as a tool to study ZMPSTE24 activity and confirm that ZMPSTE24 is required for prelamin A processing.

The most studied progeria-associated mutations involve the removal of ZMPSTE24 recognition sites, causing the accumulation around the nuclear periphery of the constitutively farnesylated protein progerin. However, it is unknown whether *LMNA* missense point mutations found in atypical progeria syndromes or other laminopathies exhibit a similar prelamin A accumulation. Therefore, we generated plasmids containing 3XFLAG-tagged human *LMNA* cDNA with a subset of point mutations found in progeria and congenital muscular dystrophy (Table S1). In particular, we focused on the missense point mutations R527C, T528M, M540T, K542N, G608S and R644H localized in the tail region of the human lamin A (Fig. S3D, Table S1). These transgenes, under the control of low expressing promoters in MEFs (Fig. S4B-C).^60^ were transiently expressed in *Lmna*^−/−^ MEFs and the prelamin A:lamin A ratio was assessed. Immunofluorescence analysis of the transiently expressed proteins showed that all lamin A variants localized at the nuclear envelope similarly to the wild type protein (Fig. S4D-I). Semi-quantitative immunblotting revealed that the G608S mutation alone was capable of causing a consistent prelamin A accumulation (Fig. 5E-F). Interestingly, M540T and K542N mutants instead exhibited reduced prelamin A levels compared to the mature version. These results suggest that the latter mutants could affect processing efficiency or protein turnover. Additionally, while R527C and T528M mutants do not show any alteration in prelamin A:lamin A ratio compared to wild type, the R644H substitution completely abolished PL-1C7 recognition of prelamin A. Prelamin A R644 is part of the PL-1C7 recognition sequence TRSYLL and thus is necessary for PL-1C7 binding. Thus, to determine whether R644H substitution affects prelamin A levels, we used the antibody 7G11 and found no alterations on the prelamin A accumulation in this mutant (Fig. 5E). These results suggest that prelamin A accumulation is not the only means by which mutations in the *LMNA* gene affect the biology of this structural protein. Given the differences in target epitopes and sensitivities, the combined side-by-side use of both PL-1C7 and 7G11 antibodies appears to be optimal for studying prelamin A expression and processing.

## Discussion

Lamin A is synthesized as a precursor, prelamin A, which has 98 unique C-terminal amino acids. The last 20 amino acids of lamin A undergo a series of post-translational modifications including isoprenylation followed by removal of the last 18 amino acids via the metalloproteinase ZMPSTE24.^15,18,56^ Here we have generated the PL-1C7 antibody and shown that it specifically recognizes the intact prelamin A sequence by targeting the ZMPSTE24-recognition sequence. The antibody allows prelamin A quantification via intracellular flow cytometry or immunoblotting, as well immunostaining to determine its cellular localization. Using this novel antibody along with fluorescent microscopy, we observed that prelamin A accumulates in discrete, punctate regions around the nuclear periphery, while the mature lamin A is more homogenously distributed. Importantly, PL-1C7 detects the diffuse nucleoplasmic accumulation of endogenous prelamin A induced by FTinh, but does not detect large clumps of prelamin A upon FTinh treatment, unless the cell line is expressing a GFP-prelamin A fusion. Finally, using a selected battery of laminopathy-associated lamin A mutants, *Zmpste24*^−/−^ null cells and ZMPSTE24 inhibitors, we found that PL-1C7 can distinguish among different mutant effects on the protein levels of prelamin A.

In the last several years, the study of the multistep processing of prenylated proteins has revealed how cells fine-tune protein localization, accumulation and maturation in normal cells and how these processes are disrupted in mutant cells, including laminopathies.^15,19,25,27,30,31,61,62^ However, the study of basic aspects of lamin biology has been limited to a degree by the availability of specific tools. As monoclonal antibodies are generally the desired immunochemical standard for cell biology, we produced a monoclonal antibody that recognizes prelamin A specifically at the sequence cleaved by ZMPSTE24 to produce the mature lamin A. Previously, rabbit polyclonal serum against prelamin A had been generated using the peptide LLGNSSPRTQSPQN, which is located downstream of ZMPSTE24 cleavage sequence. In addition to the potential variability usually generated by different animals, this polyclonal antibody cannot distinguish between the prelamin A and the farnesylated peptide generated by ZMPSTE24 upon lamin A cleavage.^17^ In a similar approach to generate a polyclonal serum against prelamin A and its farnesylated form, the synthetic peptides LLGNSSPRTQSPQNCSIM and LLGNSSPRTQSPQNC-Farnesyl were used as antigens; however, again, these polyclonal antibodies failed to distinguish prelamin A from the peptide generated by ZMPSTE24 in immunofluorescence experiments.^63^ Additionally, rat monoclonal antibody 7G11 was produced against the same region from the mouse prelamin A sequence (LLGNSSPRSQSSQNCSIM) and was shown to detect both mouse and human prelamin A, but not mature lamin A, when prelamin A accumulation was triggered via chemical or genetic approaches.^40,41^ However, we show here that this antibody also binds to the carboxy fragment generated by ZMPSTE24 cleavage. Unlike these antibodies, the PL-1C7 monoclonal antibody described here binds preferentially to the unmodified and conserved ZMPSTE24 cleavage TRSYLL sequence, but not to either of the products generated by ZMPSTE24 cleavage of prelamin A. Biochemical and microscopy approaches reveal that PL-1C7 antibody has a higher sensitivity than the 7G11 antibody in detecting low levels of prelamin A in murine cells, in particular. This difference in sensitivity could potentially be due to differing affinities, the monoclonal origin (rat vs mouse) or a specific physical constraint caused by the close proximity of the 7G11 epitope to the farnesylation site. Importantly, PL-1C7 also provides an unlimited supply with which to study not only prelamin A biology, but also ZMPSTE24 activity and function, which is difficult to evaluate with current immunological tools. Nonetheless, the 7G11 antibody may still be the tool of choice in cases where the ZMPSTE24 cleavage site is removed or mutated. Therefore, as different mutations could impact epitope recognition by these antibodies, the use of these antibodies in parallel seems optimal in studying prelamin A biology.

Lamin A plays key roles in nuclear homeostasis, thus its expression and assembly must be coordinated during cellular division and differentiation. Recently, a more complex and dynamic picture of lamin A biology has emerged, suggesting that lamins are key players in coordinating diverse nuclear functions in response to extracellular cues via the cytoskeleton.^53,64^ It has been reported that *D. melanogaster* lamin Dm0 can interact with the actin nucleator factor WASH suggesting a physical link between the machinery that regulates the cytoskeleton and the nuclear lamina.^65^ Additionally, lamin A levels are higher in tissues that possess physical elasticity (i.e. muscle), where the extracellular environment determines tissue stiffness, lamin A levels affect the physical properties of the nuclear envelope in order to compensate for the stress projected onto the nucleus.^53^ Therefore, coordinated lamin A assembly and disassembly must occur in those tissues upon extension and contraction. Our results suggest that prelamin A is localized in discrete areas of the nuclear envelope both in mouse myoblasts and fibroblasts, and its detection was possible due to the high sensitivity of the PL-1C7 antibody. Future work will determine whether these prelamin A foci respond to the polarization of the cells, ZMPSTE24 localization, nuclear pore distribution, random lamin A turnover and/or physical stress. Genome-related functions of lamins are being evaluated in this context, as it was recently suggested that chromatin tethering to the nuclear periphery is required to impart stiffness to nuclei and for attenuating the flow of chromatin inside the nucleus.^64^ Consistent with this, lack of lamin A increases chromatin dynamics within the nucleus, suggesting that lamin A is essential for the maintenance of genome organization and chromatin dynamics.^66^ Thus, as lamin A-enriched domains must maintain specific interactions with chromatin, lamin A turnover must be tightly regulated. Moreover, these results suggest that prelamin A processing and maturation must also be coordinated, given the dynamic changes in chromatin organization upon physical stress. Therefore, we believe PL-1C7 will be an important tool in investigating how prelamin A targeting and processing are coordinated in tissues with different stiffness, as well as in studies of the influence of specific *LMNA* mutations on these cellular processes.

The antibody described here is also a particularly useful tool for the study of prelamin A in progeroid laminopathies, metabolic laminopathies and lipodystrophies.^33,54,57,58,67^ Several reports support the involvement of farnesylated prelamin A in these disorders.^68^ For example, restrictive dermopathy, a more severe progeroid syndrome, is caused by a deficiency in ZMPSTE24 that results in a dramatic accumulation of prelamin A at the nuclear rim.^57,69,70^ Our results support this, as PL-1C7 antibody showed that cells lacking ZMPSTE24 expression and activity exhibit a more dramatic accumulation of prelamin A at the nuclear envelope. Thus, PL-1C7 antibody can potentially be used as a sensor for ZMPSTE24 expression or activity in order to characterize mutations or alterations in the prelamin A processing pathway. An exception would be the HGPS mutation, G608G, which results in a 50 amino acid deletion removing the ZMPSTE24 cleavage site, and producing a mutant protein (progerin) that remains farnesylated. The study of this mutant led to the suggestion that abnormal processing and accumulation of prelamin A interferes with normal lamin A functions, resulting in nuclear abnormalities.^25^ Several missense mutations in the *LMNA* gene have also been associated with atypical progeria syndromes, metabolic laminopathies and lipodystrophies, but it has been unclear whether their associated phenotypes are due to prelamin A accumulation. Here, using the PL-1C7 antibody, we provided evidence that, when the missense mutation G608S is expressed in the context of the full-length protein, prelamin A accumulation increases in comparison to the other tested lamin A variants. Interestingly, the G608S mutation, in addition to an amino acid substitution, also introduces a cryptic splicing site in exon 11 of the *LMNA* gene causing the removal of 50 amino acids, including the ZMPSTE24 site. However, the variation in the penetrance of these cryptic splice sites within the exon 11 of the lamin A gene causes the mixed production of the lamin A G608S and progerin in the same cells. While progerin accumulation has been shown to be toxic, it has been unclear whether the G608S mutation alone affects prelamin A accumulation. Our results show that indeed, a transgene expressing the lamin A G608S variant in a *Lmna*^−/−^ background also causes the accumulation of the mutant prelamin A. Therefore, specific alterations to the primary sequence of prelamin A in domains distal to the carboxyl end can potentially affect prelamin A processing, leading to accumulation of the precursor. However, the consequences of the accumulation of both prelamin A G608S and progerin in the same cells remain undetermined.

In contrast, no prelamin A accumulation was observed in mouse fibroblasts expressing a subset of human lamin A mutants associated with APS (R527C, T528M, M540T and K542N) and congenital muscular dystrophy (R644H). In fact, while no effects were observed with the mutants R527C and T528M, our data shows that mutants M540T and K542N exhibit a more efficient processing from prelamin A to lamin A. In this context, the identification of progeria-like disease phenotypes in patients with missense point mutations far away from the prelamin A farnesylation site is intriguing and suggests that the primary structure of lamin A itself, and not its farnesyl lipid anchor, is key to the pathogenesis of HGPS. Consistent with this, progeria phenotypes are observed in mice expressing a non-farnesylated progerin, suggesting that the presence of this protein and not the farnesylation anchor alter the physiology of the cells.^71^ These results thus contrast with studies where prelamin A accumulation has been suggested as the cause of the toxicity in some of these laminopathies.^63,72^ For example, increased levels of prelamin A were reported in the human mutant lamin A R482W, which is associated with Type 2 familial partial lipodystrophy, but the antibody used for the cytological characterization was a commercial polyclonal antibody generated against the lamin A carboxyl terminus, which recognizes both immature and mature lamin A.^63,72^ Therefore, the use of prelamin A specific tools will allow a better understanding these missense point mutations and their role in diverse laminopathies.

In summary, as our prelamin A-specific PL-1C7 antibody relies on the integrity of the ZMPSTE24 cleavage site, it can be used to specifically analyze prelamin A accumulation via qualitative (i.e., microscopy) or quantitative (i.e. intracellular flow cytometry) approaches. Thus, this antibody will be a particularly useful tool in investigating basic lamin A biology, as well as the role of the prelamin A in various disease states.^63,73,74^ Moreover, given the differences in target epitopes and sensitivities, the parallel use of both PL-1C7 and 7G11 antibodies provides a useful tool kit for studying prelamin A expression and processing.

## Materials and methods

### Cell lines

Both mouse (C2C12 mouse myoblasts, *Zmspte24*^−/−^ and *Lmna*^−/−^ null mouse embryonic fibroblasts) and human (rhabdomyosarcoma (A-204), osteosarcoma (U-2 OS), foreskin fibroblast (HFF) and cervix adenocarcinoma (HeLa)) cell lines were cultured in Dulbecco's modified Eagle medium (Thermo fisher scientific, Cat No. 12491-015) containing 10% Fetal Bovine Serum (FBS; Thermo fisher scientific, Cat No. 10437-077) and L-Glutamine at 37°C and 5% CO_2_.

### Development of GFP-Lmna inducible cell lines

To generate the *GFP-Lmna* fusion, the cDNA coding for the 665 amino acids of the full-length murine prelamin A (including the ZMPSTE24 cleavage and CAAX motif) was cloned into the pEGFP-C1 vector (Clonetech) using the *XhoI* and *BamHI* sites. The *GFP-Lmna* fusion cDNA was then transferred to an inducible-expression system based on the transposition system Tol2/TRE plasmid and Tol2 transposase.^75^ This vector was modified from its original version by introducing a Tet-On system, which is optimized for mammalian codon usage and supplemented with a TRE-Tight system allowing gene expression induction using Doxycycline (Amin and Groudine, in preparation). The plasmid containing the inducible GFP-*Lmna* transposon was co-transfected with a plasmid encoding Tol2 transposase at a 1:5 (Transposase:Transposon) ratio into *Lmna*^−/−^ MEFs or C2C12 cells with Fugene HD according to the manufacturer protocol (Promega, Cat. No. E2311). The cells were selected with puromycin (2 μg/ml; Invitrogen, Cat. no. A1113802) for 2 weeks and maintained in media supplemented with 1 μg/ml of puromycin. Doxycycline (Sigma, Cat. no. D9871) was titered and used at the lowest effective concentration (0.1 ug/ml) to achieve consistent *GFP-lmna* fusion expression (as detected by immunofluorescence microscopy) after incubation for 24 h at 37°C.

### PL-1C7 monoclonal antibody development

Murine PL-1C7 monoclonal antibody was generated at the Fred Hutchinson Antibody Technology Core Facility. Briefly, BALB/c, CD1, and Swiss Webster mice were immunized with the TRSYLLGNSSPR peptide (CHIScientific) maleimide coupled to KLH carrier protein. Following a 12-week boosting protocol, splenocytes were isolated from high titer mice, electrofused to FoxNY myelomas (BTX, Harvard Apparatus), and hybridomas secreting peptide specific antibody were identified and isolated using a ClonePixII colony picker. Antibodies from the picked clones were validated by flow cytometry using a peptide coupled cytometric bead array. The validated clone was put through 2 rounds of subcloning using the ClonePixII followed by another round of cytometric bead binding validation. The antibody PL-1C7 was further characterized and epitope-mapped to the TRSYLLGNSSPR peptide using standard enzyme-linked immunosorbent assay (ELISA) assays.

### Peptides and ELISA

The following synthetic peptides (purchased from Thermo Scientific) were used for ELISA immunoassays: pLA_WT: TRSYLLGNSSPR; pLA_1: AAAAYLLGNSSPR; pLA_2: TRSAAAGNSSPR; pLA_3: TRSYLLAAASPR; pLA_4: TRSYLLGNSAAA; pLA_Mat: GSFGDNLVTRSY; pLA_Frag: LLGNSSPRTQS. Peptides were incubated on 96-well EIA plates at 4°C for 16h. Unbound peptide was washed off with PBS and plates were blocked. ELISA was performed using an anti-mouse IgG HRP ELISA kit (KPL, Cat. no. 54-62-18) according to the manufacturer's instructions. Absorbance was read at 405 nm in an Envision ELISA reader (PerkinElmer).

### Intracellular flow cytometry

1 × 10^6^ cells were plated in 10-cm dishes and grown 12 h before Dox-induction. After 24 h of Dox-induction the cells were rinsed with PBS, detached with trypsin (Thermo fisher scientific, Cat no. R-001-100) and then spun at 400 g for 5 min. Cells were fixed in 3% formaldehyde, 2% sucrose in PBS at room temperature for 10 min. Fixed cells were washed with PBS 3 times and permeabilized with 0.2% Triton/PBS. Cells were again washed 3 times with PBS for 5 min each, and then resuspended in blocking buffer (2% BSA, 4% FBS in PBS) for 3 h at 4°C. These cells were incubated with the primary antibodies in blocking buffer for 12 h at 4°C. Cells were washed 3 times with 0.2% Tween in PBS and incubated with secondary antibodies for 30 min at 4°C. Cells were again washed 3 times with PBS, resuspended in blocking buffer and analyzed by flow cytometry using a BD Canto cytometer (BD Biosciences). 1 × 10^4^ events were analyzed for each population of interest. Primary antibodies included: monoclonal anti-prelamin A clone PL-1C7 (antibody concentration: 1:5 dilution for supernatant or 1 μg/sample for purified antibody; FHCRC); goat polyclonal Lamin B antibody (1:200 dilution, Santa Cruz Biotechnologies, sc-6217); rabbit Lamin A/C antibody (1:10 dilution; Abcam, ab133256). Secondary antibodies included: donkey anti-mouse IgG Alexa Fluor-488 (1:500 dilution; Invitrogen Life Technologies, Cat no. A21202), donkey anti-rabbit IgG Alexa fluor-594 (1:500 dilution; Invitrogen Life Technologies, Cat no. A21203), or donkey anti-goat IgG Alexa fluor-647 (1:500 dilution, Cat. no. A31571).

### Inhibitor treatment

Farnesyl transferase inhibitor Lonafarnib was used at 3.2 μM. The inhibitor was added to cells 4 h after Dox induction. Cells were prepared for microcopy analysis as described below. For inhibition of ZMPSTE24 activity, C2C12 myoblasts were cultured on gelatinized slides and treated with the HIV protease inhibitor Indinavir for 24 h (IDV, diluted in H_2_0, 20 μM) kindly provided by the McElrath Lab (Fred Hutchinson Cancer Research Center). Cells were washed, fixed with paraformaldehyde and processed as described above.

### Western blot

Nuclear protein fractions were purified as described.^76^ Briefly, pelleted cells were resuspended in NP-40/sucrose buffer (0.32 M Sucrose, 3 mM CaCl_2_, 2 mM MgCl_2_, 0.1 mM EDTA and 1.5% NP-40, protease inhibitors) and allowed to lyse on ice for 5 min. Nuclei were pelleted at 1500 g and washed once with the sucrose buffer without NP-40 and pelleted. For protein extraction, nuclei were resuspended in Radioimmunoprecipitation assay buffer (RIPA) buffer and sonicated with 5 pulses for 5 sec each (40% power, Fisher scientific sonic dismembranator, Model 505). Sonicated lysate was incubated on ice for 10 min and protein concentrations were measured using a BCA assay kit (Pierce, Cat. No. 23238). 50 μg of protein were resuspended in SDS loading buffer (Novex life technologies, Cat. no. N00007) and resolved on a 4–20% acrylamide gel (BIORAD, Cat. no. 456–1094) at 100 Volts for 1 h. The proteins were transferred to a PVDF membrane (Life technologies, Cat no. 88518) in 20% Methanol TRIS-Glycine-SDS buffer at 100 Volts for 2 h. After transfer, the membranes were blocked in LI-COR blocking buffer (LI-COR Odyssey, Cat. no. 927–40000) or BSA blocking solution (5% BSA, 0.1 % Tween 20 in TBS) overnight at 4°C and then blotted with the primary antibodies for 2 h at RT. The membranes were washed 3 times with TBST (0.1 % Tween 20, Tris-buffered saline -TBS-) and incubated with the secondary antibodies in blocking buffer for 1 h at RT. Membranes were then washed and the signal was detected by chemiluminescence or by using a LI-COR Odyssey infrared imaging system (LI-COR Biosciences). For immunoblotting of transient transfected cells, total extracts were prepared by resuspending the cells in RIPA buffer with proteases inhibitors. Extracts were incubated on ice for 10 min and sonicated with 30 sec pulses 5 times. 50 mg of total protein were resolved in 4–12% acrylamide gel (Invitrogen Life technology, Cat no. NP0321BOX), transferred to a nitrocellulose membrane and blocked overnight with Odyssey blocking buffer-TBS. Antibody incubation was performed as described before. An infrared imaging system was used to quantify prelamin levels as the ratios: prelamin A/(lamin A+prelamin A). Transfection normalization was performed by adjusting the signals to the highest value in each channel. Three normalized ratios of 2 biological replicates were averaged and p values were obtained with a 2-tailed Student's t-Test (heteroscedastic). Membranes were stripped and re-blotted with anti-prelamin A 7G11 and anti-β-actin antibodies and analyzed by chemiluminescence. Primary antibodies used: rabbit IgG anti-GFP (1:1000 dilution; Invitrogen Life technologies); goat polyclonal anti-PARP (1:1000 dilution; Santa Cruz, sc-9935,), monoclonal anti-prelamin A PL-1C7 (1μg/ml -purified antibody-; FHCRC), rat monoclonal anti-prelamin A 7G11 (1μg/ml; Millipore; Cat no. MABT345), mouse anti-β-actin (1:1000; Cell Signaling technology; cat no. 3700S), rabbit anti-lamin A/C (1:200 dilution; Abcam, ab108595), rabbit anti-FLAG antibody (1:1000; Thermo Fisher Scientific PA1-984B). Secondary antibodies used: donkey anti-rabbit IRDYE 680CW and anti-mouse IRDYE 800CW (1:15000 dilution; LI-COR Biosciences, Cat. No. 926–68073 and 926–32212) antibodies, or HRP-conjugated anti rabbit, goat and mouse (1:15000 dilution, Jackson Immunoresearch, 711-036-1552, 705-036-147, 715-036-150).

### Immunostaining

Cultured C2C12 myoblasts or MEF cells were cultured in chambered slides (Nunc Lab-Tek, Cat. No. 177399). The slides were washed with PBS and the cells fixed in 4% paraformaldehyde/PBS at RT for 10 min. *GFP-Lmna* MEFs were fixed after 24 h of Dox induction (0.1 μM). After fixation the cells were washed with PBS 3 times, followed by permeabilization with 0.2% Triton/PBS for 10 min, then washed again 3 times with PBS and then blocked with PAT buffer (1% BSA, 0.1% Tween 20 in PBS) for 30 min. The slides were incubated with the primary antibody for 2 h at RT, washed again and then incubated with secondary antibodies in PAT buffer at RT for 1 h. The primary antibodies used include: mouse monoclonal anti-prelamin A PL-1C7 antibody (1:100 dilution for supernatant or 1μg/ml for purified antibody; FHCRC); rat monoclonal anti-prelamin A 7G11 (1μg/ml; Millipore), goat polyclonal anti-Lamin B antibody (1:1000 dilution; Santa Cruz Biotechnologies, sc-6217); rabbit anti-lamin A/C antibody (1:200 dilution; Abcam, ab108595). The secondary antibodies include: donkey anti-mouse IgG Alexa Fluor-488 (1:200 dilution; Invitrogen Life Technologies, Cat no. A21202), donkey anti-rabbit IgG Alexa fluor-594 (1:200 dilution; Invitrogen Life Technologies, Cat no. A21207), donkey anti-mouse IgG Alexa fluor-594 (1:200 dilution; Invitrogen Life Technologies, Cat no. A21203), donkey anti-rat IgG Alexa fluor 595(1:200 dilution; Invitrogen Life Technologies, Cat no A21209) or donkey anti-goat IgG Alexa fluor-647 (1:200 dilution, Cat. no. A31571.) The slides were mounted in Slow Fade Gold with DAPI (Invitrogen Life technologies, Cat No. S36940).

For methanol fixation, C2C12 wild type myoblasts were grown, fixed and permeabilized as described.^77^ Briefly, 50,000 cells were grown on chambered slides as above, the slides were washed with PBS and incubated in cold methanol for 10 min at −20°C. Cells were then washed with PBS 3 times before blocking with PAT buffer (1% BSA, 0.1% Tween 20 in PBS) for 30 min and subsequently incubated with the primary and secondary antibody as described above.

To validate endogenous prelamin A detection with PL-1C7 antibody in MEF, *Lmna*^−/−^ null and wild type MEFs were trypsinized, counted and equal numbers of each genotype were mixed together and co-culture on gelatinized slides. The slides were fixed and immunostained against prelamin A, lamin A and lamin B as described above.

To analyze lamin A mutants, 3XFLAG-tagged wild-type human *LMNA* cDNA containing the ZMPSTE24 cleavage and CAAX motif was obtained from GeneCopoeia (ORF expression vector, EX-Z3407-M12). Six previously reported lamin A mutants (Table S1) were generated by replacing the carboxyl terminus with synthesized mutation-containing fragments using the Gibson assembly cloning kit (NEB, Cat. #E5510S). The plasmids were independently transfected in *Lmna*^−/−^ MEFs (1 × 10^5^ cells/transfection) using a Lipofectamine 3000 kit according to the manufacturer's protocol (ThermoFisher Scientific, Cat. No. L3000008). For western blotting, 10 μg of plasmid were transfected in 5 × 10^6^ *Lmna*^−/−^ null MEFs growing in 10-cm petri dish. The cells were detached with trypsin 48 h post-transfection and harvested to prepare total protein extracts for protein gel blot or transferred to chambered slides and prepared for immunodetection.

### Microscopy and image analysis

Image acquisition was performed on a DeltaVision Elite image restoration system (Applied Precision Inc.). Using a 60X objective, 38–60 optical sections were obtained using a step size of 0.2 μm in the z-axis. 1 μm projections were generated by averaging the signal of each channel. Images were deconvolved using SoftWoRX (Applied Precision Inc.,) and processed with ImageJ (ImageJ, U. S. National Institutes of Health, Bethesda, Maryland, USA, http://imagej.nih.gov/ij/, 1997–2014). To generate publication quality pictures only contrast and brightness were adjusted. The quantification of signal continuity across the nuclear envelope for prelamin A and lamin A/C was performed using fluorescence intensity linescan profiles and a newly written ‘relative Fluctuation index’ algorithm. Fluorescence intensity linescan plots across a line drawn over a segment of the nuclear envelope of representative cells were created for prelamin A and lamin A/C signal channels using the ImageJ tool, Plot Profile (Image J, National Institute of Health, Bethesda, MD, USA). To compute the ‘fluctuation index’, the deconvolved 3D images were exported as 16 bit tiff stacks. From each 3D image stack, a 2D maximum intensity projection (MIP) images for the DAPI and lamin-stained channels was derived. Nuclei boundaries and their corresponding lamin-labeled contours were derived from the respective 2D images using a 2-step procedure. First, an initial boundary was estimated using the Otsu intensity-based thresholding method.^78^ The boundary contour for each nucleus was subsequently refined using the gradient vector field (GVF) based parametric active contour segmentation method.^79^ Parameters for the active contour were empirically estimated from the image data, and 75 iterations were performed to obtain the final nucleus boundary. The ‘fluctuation index’ for each nucleus was then computed from the 2 channels (channel 1: reference Lamin B and channel 2: experimental, either lamin A/C or prelamin A) of the MIP image as follows: 1) The first derivative of the pixel intensities along the nuclear envelope contour in both channels was computed and normalized. 2) The ratio between the corresponding derivative values in both channels (Ichannel1/Ichannel2, Ichannel2/Ichannel1) was computed at every pixel along the Lamin contour and normalized again for a final value range of 0 to 1. Potential errors such as ‘divide by zero’ were corrected at this stage. 3) The fraction of pixels with ratios above 0.15 were computed for both cases, expressed as a percentage, and added.

## Supplementary Material

2015NUCLEUS0062R-s02.pdf

## References

[1] Dwyer N, Blobel G. A modified procedure for the isolation of a pore complex-lamina fraction from rat liver nuclei. J Cell Biol 1976; 70:581-91; PMID:986398; http://dx.doi.org/10.1083/jcb.70.3.581 986398 PMC2109848

[2] Schreiber KH, Kennedy BK. When lamins go bad: nuclear structure and disease. Cell 2013; 152:1365-75; PMID:23498943; http://dx.doi.org/10.1016/j.cell.2013.02.015 23498943 PMC3706202

[3] Gruenbaum Y, Foisner R. Lamins: nuclear intermediate filament proteins with fundamental functions in nuclear mechanics and genome regulation. Annu Rev Biochem 2015; 84:131-64; PMID:25747401; http://dx.doi.org/10.1146/annurev-biochem-060614-034115 25747401

[4] Burke B, Stewart CL. Functional architecture of the cell's nucleus in development, aging, and disease. Curr Top Dev Biol 2014; 109:1-52; PMID:24947235; http://dx.doi.org/10.1016/B978-0-12-397920-9.00006-8 24947235

[5] Davidson PM, Lammerding J. Broken nuclei – lamins, nuclear mechanics, and disease. Trends Cell Biol 2014; 24:247-56; PMID:24309562; http://dx.doi.org/10.1016/j.tcb.2013.11.004 24309562 PMC3972295

[6] Lin F, Worman HJ. Structural organization of the human gene (LMNB1) encoding nuclear lamin B1. Genomics 1995; 27:230-6; PMID:7557986; http://dx.doi.org/10.1006/geno.1995.1036 7557986

[7] Biamonti G, Giacca M, Perini G, Contreas G, Zentilin L, Weighardt F, Guerra M, Valle Della G, Saccone S, Riva S. The gene for a novel human lamin maps at a highly transcribed locus of chromosome 19 which replicates at the onset of S-phase. Mol Cell Biol 1992; 12:3499-506; PMID:1630457; http://dx.doi.org/10.1128/MCB.12.8.3499 1630457 PMC364599

[8] Weber K, Plessmann U, Traub P. Protein chemical analysis of purified murine lamin B identifies two distinct polypeptides B1 and B2. FEBS Lett 1990; 261:361-4; PMID:2311764; http://dx.doi.org/10.1016/0014-5793(90)80592-7 2311764

[9] Holtz D, Tanaka RA, Hartwig J, McKeon F. The CaaX motif of lamin A functions in conjunction with the nuclear localization signal to target assembly to the nuclear envelope. Cell 1989; 59:969-77; PMID:2557160; http://dx.doi.org/10.1016/0092-8674(89)90753-8 2557160

[10] Kitten GT, Nigg EA. The CaaX motif is required for isoprenylation, carboxyl methylation, and nuclear membrane association of lamin B2. J Cell Biol 1991; 113:13-23; PMID:2007618; http://dx.doi.org/10.1083/jcb.113.1.13 2007618 PMC2288919

[11] Strelkov SV, Schumacher J, Burkhard P, Aebi U, Herrmann H. Crystal structure of the human lamin A coil 2B dimer: implications for the head-to-tail association of nuclear Lamins. J Mol Biol 2004; 343:1067-80; PMID:15476822; http://dx.doi.org/10.1016/j.jmb.2004.08.093 15476822

[12] Wei MG, Tong XJ, Bin Chen, Zhang B, Liu ZF, Ding MX, Zhai ZH Assembly of lamins in vitro. Cell Res 1996; 6:11-22; http://dx.doi.org/10.1038/cr.1996.2

[13] Weber K, Plessmann U, Traub P. Maturation of nuclear lamin A involves a specific carboxy-terminal trimming, which removes the polyisoprenylation site from the precursor; implications for the structure of the nuclear lamina. FEBS lett 1989; 257:411-4; PMID:2583287; http://dx.doi.org/10.1016/0014-5793(89)81584-4 2583287

[14] Beck LA, Hosick TJ, Sinensky M Isoprenylation is required for the processing of the lamin A precursor. J Cell Bio 1990 ; 119:1489-99. PMID: 2335559.9030603 10.1083/jcb.110.5.1489 PMC2200179 2335559

[15] Kilic F, Dalton MB, Burrell SK, Mayer JP, Patterson SD, Sinensky M. In vitro assay and characterization of the farnesylation-dependent prelamin A endoprotease. J Biol Chem 1997; 272:5298-304; PMID:9030603; http://dx.doi.org/10.1074/jbc.272.8.5298 9030603

[16] Hennekes H, Nigg EA. The role of isoprenylation in membrane attachment of nuclear lamins. A single point mutation prevents proteolytic cleavage of the lamin A precursor and confers membrane binding properties. J Cell Sci 1994; 107 (Pt 4):1019-29; PMID:8056827 8056827 10.1242/jcs.107.4.1019

[17] Sinensky M, Fantle K, Dalton M. An antibody which specifically recognizes prelamin A but not mature lamin A: application to detection of blocks in farnesylation-dependent protein processing. Cancer Res 1994; 54:3229-32; PMID:8205544 8205544

[18] Pendás AM, Zhou Z, Cadiñanos J, Freije JMP, Wang J, Hultenby K, Astudillo A, Wernerson A, Rodríguez F, Tryggvason K, et al. Defective prelamin A processing and muscular and adipocyte alterations in Zmpste24 metalloproteinase–deficient mice. Nat Genet 2002; 31:94-9 11923874 10.1038/ng871

[19] Sinensky M, Fantle K, Trujillo M, McLain T, Kupfer A, Dalton M. The processing pathway of prelamin A. J Cell Sci 1994; 107 (Pt 1):61-7; PMID:8175923 8175923 10.1242/jcs.107.1.61

[20] Fong LG, Ng JK, Lammerding J, Vickers TA, Meta M, Coté N, Gavino B, Qiao X, Chang SY, Young SR, et al. Prelamin A and lamin A appear to be dispensable in the nuclear lamina. J Clin Invest 2006; 116:743-52; PMID:16511604; http://dx.doi.org/10.1172/JCI27125 16511604 PMC1386109

[21] Bonne G, Di Barletta MR, Varnous S, Bécane HM, Hammouda EH, Merlini L, Muntoni F, Greenberg CR, Gary F, Urtizberea JA, et al. Mutations in the gene encoding lamin A/C cause autosomal dominant Emery-Dreifuss muscular dystrophy. Nat Genet 1999; 21:285-8; PMID:10080180; http://dx.doi.org/10.1038/6799 10080180

[22] Muchir A. Identification of mutations in the gene encoding lamins A/C in autosomal dominant limb girdle muscular dystrophy with atrioventricular conduction disturbances (LGMD1B). Hum Mol Genet 2000; 9:1453-9; PMID:10814726; http://dx.doi.org/10.1093/hmg/9.9.1453 10814726

[23] Shackleton S, Lloyd DJ, Jackson SN, Evans R, Niermeijer MF, Singh BM, Schmidt H, Brabant G, Kumar S, Durrington PN, et al. LMNA, encoding lamin A/C, is mutated in partial lipodystrophy. Nat Genet 2000; 24:153-6; PMID:10655060; http://dx.doi.org/10.1038/72807 10655060

[24] Novelli G, Muchir A, Sangiuolo F, Helbling-Leclerc A, D'Apice MR, Massart C, Capon F, Sbraccia P, Federici M, Lauro R, et al. Mandibuloacral dysplasia is caused by a mutation in LMNA-encoding lamin A/C. Am J Hum Genet 2002; 71:426-31; PMID:12075506; http://dx.doi.org/10.1086/341908 12075506 PMC379176

[25] Eriksson M, Brown WT, Gordon LB, Glynn MW, Singer J, Scott L, Erdos MR, Robbins CM, Moses TY, Berglund P, et al. Recurrent de novo point mutations in lamin A cause Hutchinson-Gilford progeria syndrome. Nature 2003; 423:293-8; PMID:12714972; http://dx.doi.org/10.1038/nature01629 12714972 PMC10540076

[26] Capell BC, Erdos MR, Madigan JP, Fiordalisi JJ, Varga R, Conneely KN, Gordon LB, Der CJ, Cox AD, Collins FS. Inhibiting farnesylation of progerin prevents the characteristic nuclear blebbing of Hutchinson-Gilford progeria syndrome. Proc Natl Acad Sci USA 2005; 102:12879-84; PMID:16129833; http://dx.doi.org/10.1073/pnas.0506001102 16129833 PMC1200293

[27] Goldman RD, Shumaker DK, Erdos MR, Eriksson M, Goldman AE, Gordon LB, Gruenbaum Y, Khuon S, Mendez M, Varga R, et al. Accumulation of mutant lamin A causes progressive changes in nuclear architecture in Hutchinson-Gilford progeria syndrome. Proc Natl Acad Sci USA 2004; 101:8963-8; PMID:15184648; http://dx.doi.org/10.1073/pnas.0402943101 15184648 PMC428455

[28] Barthelemy F, Navarro C, Fayek R, Da Silva N, Roll P, Sigaudy S, Oshima J, le Bonne GE, Papadopoulou-Legbelou K, Evangeliou AE, et al. Truncated prelamin A expression in HGPS-like patients: a transcriptional study. Euro J Hum Genet 2015; 23(8):1051-61; PMID:25649378; http://dx.doi.org/10.1038/ejhg.2014.239 25649378 PMC4795109

[29] Dechat T, Shimi T, Adam SA, Rusinol AE, Andres DA, Spielmann HP, Sinensky MS, Goldman RD. Alterations in mitosis and cell cycle progression caused by a mutant lamin A known to accelerate human aging. Proc Natl Acad Sci USA 2007; 104:4955-60; PMID:17360326; http://dx.doi.org/10.1073/pnas.0700854104 17360326 PMC1829246

[30] Young SG, Meta M, Yang SH, Fong LG. Prelamin A farnesylation and progeroid syndromes. J Biol Chem 2006; 281:39741-5; PMID:17090536; http://dx.doi.org/10.1074/jbc.R600033200 17090536

[31] Cao K, Capell BC, Erdos MR, Djabali K, Collins FS. A lamin A protein isoform overexpressed in Hutchinson-Gilford progeria syndrome interferes with mitosis in progeria and normal cells. Proc Natl Acad Sci USA 2007; 104:4949-54; PMID:17360355; http://dx.doi.org/10.1073/pnas.0611640104 17360355 PMC1821129

[32] Fong LG, Frost D, Meta M, Qiao X, Yang SH, Coffinier C, Young SG. A protein farnesyltransferase inhibitor ameliorates disease in a mouse model of progeria. Science 2006; 311:1621-3; PMID:16484451; http://dx.doi.org/10.1126/science.1124875 16484451

[33] Glynn MW. Incomplete processing of mutant lamin A in Hutchinson-Gilford progeria leads to nuclear abnormalities, which are reversed by farnesyltransferase inhibition. Hum Mol Genet 2005; 14:2959-69; PMID:16126733; http://dx.doi.org/10.1093/hmg/ddi326 16126733

[34] Verstraeten VLRM, Verstraeten VLRM, Peckham LA, Peckham LA, Olive M, Olive M, Capell BC, Capell BC, Collins FS, Collins FS, et al. Protein farnesylation inhibitors cause donut-shaped cell nuclei attributable to a centrosome separation defect. Proc Natl Acad Sci USA 2011; 108:4997-5002; PMID:21383178; http://dx.doi.org/10.1073/pnas.1019532108 21383178 PMC3064351

[35] Lutz RJ, Trujillo MA, Denham KS, Wenger L, Sinensky M. Nucleoplasmic localization of prelamin A: implications for prenylation-dependent lamin A assembly into the nuclear lamina. Proc Natl Acad Sci USA 1992; 89:3000-4; PMID:1557405; http://dx.doi.org/10.1073/pnas.89.7.3000 1557405 PMC48791

[36] Dalton MB, Fantle KS, Bechtold HA, DeMaio L, Evans RM, Krystosek A, Sinensky M. The farnesyl protein transferase inhibitor BZA-5B blocks farnesylation of nuclear lamins and p21ras but does not affect their function or localization. Cancer Res 1995; 55:3295-304; PMID:7614464 7614464

[37] Kieran MW, Gordon L, Kleinman M. New approaches to progeria. Pedriatics 2007; 120:834-41; PMID:17908771; http://dx.doi.org/10.1542/peds.2007-1356 17908771

[38] Gordon LB, Massaro J, D'Agostino RB, Campbell SE, Brazier J, Brown WT, Kleinman ME, Kieran MW, Progeria clinical trials collaborative. Impact of farnesylation inhibitors on survival in Hutchinson-Gilford progeria syndrome. Circulation 2014; 130:27-34; PMID:24795390; http://dx.doi.org/10.1161/CIRCULATIONAHA.113.008285 24795390 PMC4082404

[39] Gordon LB, Kleinman ME, Miller DT, Neuberg DS, Giobbie-Hurder A, Gerhard-Herman M, Smoot LB, Gordon CM, Cleveland R, Snyder BD, et al. Clinical trial of a farnesyltransferase inhibitor in children with Hutchinson–Gilford progeria syndrome. Proc Natl Acad Sci USA 2012; 109:16666-71; PMID:23012407; http://dx.doi.org/10.1073/pnas.1202529109 23012407 PMC3478615

[40] Lee R, Chang SY, Trinh H, Tu Y, White AC, Davies BSJ, Bergo MO, Fong LG, Lowry WE, Young SG. Genetic studies on the functional relevance of the protein prenyltransferases in skin keratinocytes. Hum Mol Genet 2010; 19:1603-17; PMID:20106865; http://dx.doi.org/10.1093/hmg/ddq036 20106865 PMC2846164

[41] Davies BSJ, Barnes RH, Tu Y, Ren S, Andres DA, Spielmann HP, Lammerding J, Wang Y, Young SG, Fong LG. An accumulation of non-farnesylated prelamin A causes cardiomyopathy but not progeria. Hum Mol Genet 2010; 19:2682-94; PMID:20421363; http://dx.doi.org/10.1093/hmg/ddq158 20421363 PMC2883346

[42] Varela I, Pereira S, Ugalde AP, Navarro CL, Suárez MF, Cau P, Cadiñanos J, Osorio FG, Foray N, Cobo J, et al. Combined treatment with statins and aminobisphosphonates extends longevity in a mouse model of human premature aging. Nature Medicine 2008; 14:767-72; PMID:18587406; http://dx.doi.org/10.1038/nm1786 18587406

[43] Garg A, Subramanyam L, Agarwal AK, Simha V, Levine B, D'Apice MR, Novelli G, Crow Y. Atypical progeroid syndrome due to heterozygous missense LMNA mutations. J Clin Endocrinol & Metab 2009; 94:4971-83; PMID:19875478; http://dx.doi.org/10.1210/jc.2009-0472 19875478 PMC2795646

[44] Chen L, Lee L, Kudlow BA, Santos Dos HG, Sletvold O, Shafeghati Y, Botha EG, Garg A, Hanson NB, Martin GM, et al. LMNA mutations in atypical Werner's syndrome. Lancet 2003; 362:440-5; PMID:12927431; http://dx.doi.org/10.1016/S0140-6736(03)14069-X 12927431

[45] Doh YJ, Kim HK, Jung ED, Choi SH, Kim JG, Kim BW, Lee IK. Novel LMNA gene mutation in a patient with Atypical Werner's Syndrome. Korean J Intern Med 2009; 24:68-72; PMID:19270485; http://dx.doi.org/10.3904/kjim.2009.24.1.68 19270485 PMC2687649

[46] Moulson CL, Fong LG, Gardner JM, Farber EA, Go G, Passariello A, Grange DK, Young SG, Miner JH. Increased progerin expression associated with unusual LMNAmutations causes severe progeroid syndromes. Human Mutation 2007; 28:882-9; PMID:17469202; http://dx.doi.org/10.1002/humu.20536 17469202

[47] Csoka AB. Novel lamin A/C gene (LMNA) mutations in atypical progeroid syndromes. J Med Genet 2004; 41:304-8; PMID:15060110; http://dx.doi.org/10.1136/jmg.2003.015651 15060110 PMC1735741

[48] Verstraeten VLRM, Broers JLV, van Steensel MAM, Zinn-Justin S, Ramaekers FCS, Steijlen PM, Kamps M, Kuijpers HJH, Merckx D, Smeets HJM, et al. Compound heterozygosity for mutations in LMNA causes a progeria syndrome without prelamin A accumulation. Hum Mol Genet 2006; 15:2509-22; PMID:16825282; http://dx.doi.org/10.1093/hmg/ddl172 16825282

[49] Olive M, Harten I, Mitchell R, Beers JK, Djabali K, Cao K, Erdos MR, Blair C, Funke B, Smoot L, et al. Cardiovascular pathology in Hutchinson-Gilford progeria: correlation with the vascular pathology of aging. Arterioscler Thromb Vasc Biol 2010; 30:2301-9; PMID:20798379; http://dx.doi.org/10.1161/ATVBAHA.110.209460 20798379 PMC2965471

[50] Sullivan T, Escalante-Alcalde D, Bhatt H, Anver M, Bhat N, Nagashima K, Stewart CL, Burke B. Loss of A-type lamin expression compromises nuclear envelope integrity leading to muscular dystrophy. J Cell Biol 1999; 147:913-20; PMID:10579712; http://dx.doi.org/10.1083/jcb.147.5.913 10579712 PMC2169344

[51] Toth JI, Yang SH, Qiao X, Beigneux AP, Gelb MH, Moulson CL, Miner JH, Young SG, Fong LG. Blocking protein farnesyltransferase improves nuclear shape in fibroblasts from humans with progeroid syndromes. Proc Natl Acad Sci USA 2005; 102:12873-8; PMID:16129834; http://dx.doi.org/10.1073/pnas.0505767102 16129834 PMC1193538

[52] Dittmer TA, Misteli T. The lamin protein family. Genome Biol 2011; 12:222; PMID:21639948; http://dx.doi.org/10.1186/gb-2011-12-5-222 21639948 PMC3219962

[53] Swift J, Ivanovska IL, Buxboim A, Harada T, Dingal PCDP, Pinter J, Pajerowski JD, Spinler KR, Shin J-W, Tewari M, et al. Nuclear lamin-A scales with tissue stiffness and enhances matrix-directed differentiation. Science 2013; 341:1240104-15; PMID:23990565; http://dx.doi.org/10.1126/science.1240104 23990565 PMC3976548

[54] Capanni C, Mattioli E, Columbaro M, Lucarelli E, Parnaik VK, Novelli G, Wehnert M, Cenni V, Maraldi NM, Squarzoni S, et al. Altered pre-lamin A processing is a common mechanism leading to lipodystrophy. Hum Mol Genet 2005; 14:1489-502; PMID:15843404; http://dx.doi.org/10.1093/hmg/ddi158 15843404

[55] Leung GK, Schmidt WK, Bergo MO, Gavino B, Wong DH, Tam A, Ashby MN, Michaelis S, Young SG. Biochemical studies of Zmpste24-deficient mice. J Biol Chem 2001; 276:29051-8; PMID:11399759; http://dx.doi.org/10.1074/jbc.M102908200 11399759

[56] Bergo MO, Gavino B, Ross J, Schmidt WK, Hong C, Kendall LV, Mohr A, Meta M, Genant H, Jiang Y, et al. Zmpste24 deficiency in mice causes spontaneous bone fractures, muscle weakness, and a prelamin A processing defect. Proc Natl Acad Sci USA 99:13049-54; PMID:12235369; http://dx.doi.org/10.1073/pnas.192460799 12235369 PMC130584

[57] Navarro CL, De Sandre-Giovannoli A, Bernard R, Boccaccio I, Boyer A, Geneviève D, Hadj-Rabia S, Gaudy-Marqueste C, Smitt HS, Vabres P, et al. Lamin A and ZMPSTE24 (FACE-1) defects cause nuclear disorganization and identify restrictive dermopathy as a lethal neonatal laminopathy. Hum Mol Genet 2004; 13:2493-503; PMID:15317753; http://dx.doi.org/10.1093/hmg/ddh265 15317753

[58] Caron M, Auclair M, Donadille B, Béréziat V, Guerci B, Laville M, Narbonne H, Bodemer C, Lascols O, Capeau J, et al. Human lipodystrophies linked to mutations in A-type lamins and to HIV protease inhibitor therapy are both associated with prelamin A accumulation, oxidative stress and premature cellular senescence. Cell Death and Differ 2007; 14:1759-67; PMID:17612587; http://dx.doi.org/10.1038/sj.cdd.4402197 17612587

[59] Coffinier C, Hudon SE, Farber EA, Chang SY, Hrycyna CA, Young SG, Fong LG. HIV protease inhibitors block the zinc metalloproteinase ZMPSTE24 and lead to an accumulation of prelamin A in cells. Proc Natl Acad Sci USA 2007; 104:13432-7; PMID:17652517; http://dx.doi.org/10.1073/pnas.0704212104 17652517 PMC1948915

[60] Qin JY, Zhang L, Clift KL, Hulur I, Xiang AP, Ren B-Z, Lahn BT. Systematic comparison of constitutive promoters and the doxycycline-inducible promoter. PLoS One 2010; 5:10611-4; PMID:20485554; http://dx.doi.org/10.1371/journal.pone.0010611 20485554 PMC2868906

[61] Barrowman J, Hamblet C, George CM, Michaelis S. Analysis of prelamin A biogenesis reveals the nucleus to be a CaaX processing compartment. Mol Bio Cell 2008; 19:5398-408; PMID:18923140; http://dx.doi.org/10.1091/mbc.E08-07-0704 18923140 PMC2592638

[62] Wang Y, Östlund C, Choi JC, Swayne TC, Gundersen GG, Worman HJ. Blocking farnesylation of the prelamin A variant in Hutchinson-Gilford progeria syndrome alters the distribution of A-type lamins. Nucleus 2014; 3:452-62; PMID:22895092; http://dx.doi.org/10.4161/nucl.21675 22895092 PMC3474666

[63] Dominici S, Fiori V, Magnani M, Schena E, Capanni C, Camozzi D, D'Apice MR, Le Dour C, Auclair M, Caron M, et al. Different prelamin A forms accumulate in human fibroblasts: a study in experimental models and progeria. Current Biology 2015; 25:804-10; PMID:25754639; http://dx.doi.org/10.1016/j.cub.2015.01.052 19351612

[64] Schreiner SM, Koo PK, Zhao Y, Mochrie SGJ, King MC. The tethering of chromatin to the nuclear envelope supports nuclear mechanics. Nat Comm 2015; 6: 7159-7172; PMID:26074052; http://dx.doi.org/10.1038/ncomms8159 26074052 PMC4490570

[65] Verboon JM, Rincon-Arano H, Werwie TR, Delrow JJ, Scalzo D, Nandakumar V, Groudine M, Parkhurst SM. Wash interacts with lamin and affects global nuclear organization. Current Biology 2015; 25:804-10; PMID:25754639; http://dx.doi.org/10.1016/j.cub.2015.01.052 25754639 PMC4366290

[66] Bronshtein I, Kepten E, Kanter I, Berezin S, Lindner M, Redwood AB, Mai S, Gonzalo S, Foisner R, Shav-Tal Y, et al. Loss of lamin A function increases chromatin dynamics in the nuclear interior. Nat Comms 2015; 6:1-9; PMID:26299252; http://dx.doi.org/10.1038/ncomms9044 26299252 PMC4560783

[67] Filesi I, Gullotta F, Lattanzi G, D'Apice MR, Capanni C, Nardone AM, Columbaro M, Scarano G, Mattioli E, Sabatelli P, et al. Alterations of nuclear envelope and chromatin organization in mandibuloacral dysplasia, a rare form of laminopathy. Physiol Genomics 2005; 23:150-8; PMID:16046620; http://dx.doi.org/10.1152/physiolgenomics.00060.2005 16046620

[68] Maraldi NM, Lattanzi G. Involvement of prelamin A in laminopathies. Critical reviews in eukaryotic gene expression. Crit Rev Eukaryot Gene Expr 2007; 17:317-34; PMID:17725496; http://dx.doi.org/10.1615/CritRevEukarGeneExpr.v17.i4.50 17725496

[69] Navarro CL, Cadiñanos J, De Sandre-Giovannoli A, Bernard R, Courrier S, Boccaccio I, Boyer A, Kleijer WJ, Wagner A, Giuliano F, et al. Loss of ZMPSTE24 (FACE-1) causes autosomal recessive restrictive dermopathy and accumulation of Lamin A precursors. Hum Mol Genet 2005; 14:1503-13; PMID:15843403; http://dx.doi.org/10.1093/hmg/ddi159 15843403

[70] Navarro CL, Esteves-Vieira V, Courrier S, Boyer A, Duong Nguyen T, Huong LTT, Meinke P, Schröder W, Cormier-Daire V, Sznajer Y, et al. New ZMPSTE24 (FACE1) mutations in patients affected with restrictive dermopathy or related progeroid syndromes and mutation update. Eur J Hum Genet 2014; 22:1002-11; PMID:24169522; http://dx.doi.org/10.1038/ejhg.2013.258 24169522 PMC4350588

[71] Yang SH, Andres DA, Spielmann HP, Young SG, Fong LG. Progerin elicits disease phenotypes of progeria in mice whether or not it is farnesylated. J Clin Invest 2008; 118:3291-300; PMID:18769635; http://dx.doi.org/10.1172/JCI35876 18769635 PMC2525700

[72] Araújo-Vilar D, Lattanzi G, González-Méndez B, Costa-Freitas AT, Prieto D, Columbaro M, Mattioli E, Victoria B, Martínez-Sánchez N, Ramazanova A, et al. Site-dependent differences in both prelamin A and adipogenic genes in subcutaneous adipose tissue of patients with type 2 familial partial lipodystrophy. J Med Genet 2009; 46:40-8; PMID:18805829; http://dx.doi.org/10.1136/jmg.2008.059485 18805829

[73] Columbaro M, Capanni C, Mattioli E, Novelli G, Parnaik VK, Squarzoni S, Maraldi NM, Lattanzi G. Rescue of heterochromatin organization in Hutchinson-Gilford progeria by drug treatment. Cell Mol Life Sci 2005; 62:2669-78; PMID:16261260; http://dx.doi.org/10.1007/s00018-005-5318-6 16261260 PMC2773834

[74] Yang SH, Bergo MO, Toth JI, Qiao X, Hu Y, Sandoval S, Meta M, Bendale P, Gelb MH, Young SG, et al. Blocking protein farnesyltransferase improves nuclear blebbing in mouse fibroblasts with a targeted Hutchinson-Gilford progeria syndrome mutation. Proc Natl Acad Sci USA 2005; 102:10291-6; PMID:16014412; http://dx.doi.org/10.1073/pnas.0504641102 16014412 PMC1174929

[75] Balciunas D, Wangensteen KJ, Wilber A, Bell J, Geurts A, Sivasubbu S, Wang X, Hackett PB, Largaespada DA, McIvor RS, et al. Harnessing a high cargo-capacity transposon for genetic applications in vertebrates. PLoS Genet 2006; 2:169-10; PMID:17096595; http://dx.doi.org/10.1371/journal.pgen.0020169 17096595 PMC1635535

[76] Rincon-Arano H, Halow J, Delrow JJ, Parkhurst SM, Groudine M. UpSET recruits HDAC complexes and restricts chromatin accessibility and acetylation at promoter regions. Cell 2012; 151:1214-28; PMID:23177352; http://dx.doi.org/10.1016/j.cell.2012.11.009 23177352 PMC3518625

[77] Caron M, Auclair M, Sterlingot H, Kornprobst M, Capeau J. Some HIV protease inhibitors alter lamin A/C maturation and stability, SREBP-1 nuclear localization and adipocyte differentiation. AIDS 2003; 17:2437-44; PMID:14600514; http://dx.doi.org/10.1097/00002030-200311210-00005 14600514

[78] Otsu NA Threshold selection method from gray-level histograms. IEEE Trans Syst, Man, Cybern 1979; 9:62-6; http://dx.doi.org/10.1109/TSMC.1979.4310076

[79] Xu C, Chenyang Xu, Prince JL. Snakes, shapes, and gradient vector flow. IEEE Trans on Image Process 1998; 7:359-69; PMID:18276256; http://dx.doi.org/10.1109/83.661186 18276256

