# Supplementary material for: Prelamin A processing, accumulation and distribution in normal cells and laminopathy disorders
Source: Nucleus. 2016 Feb 22;7(1):84–102. doi: 10.1080/19491034.2016.1150397 (PMC4916894; doi:10.1080/19491034.2016.1150397)
Supplement: 2015NUCLEUS0062R-s02.pdf [file kncl-07-01-1150397-s001.pdf]

Figure S1. Casasola et al. 2016

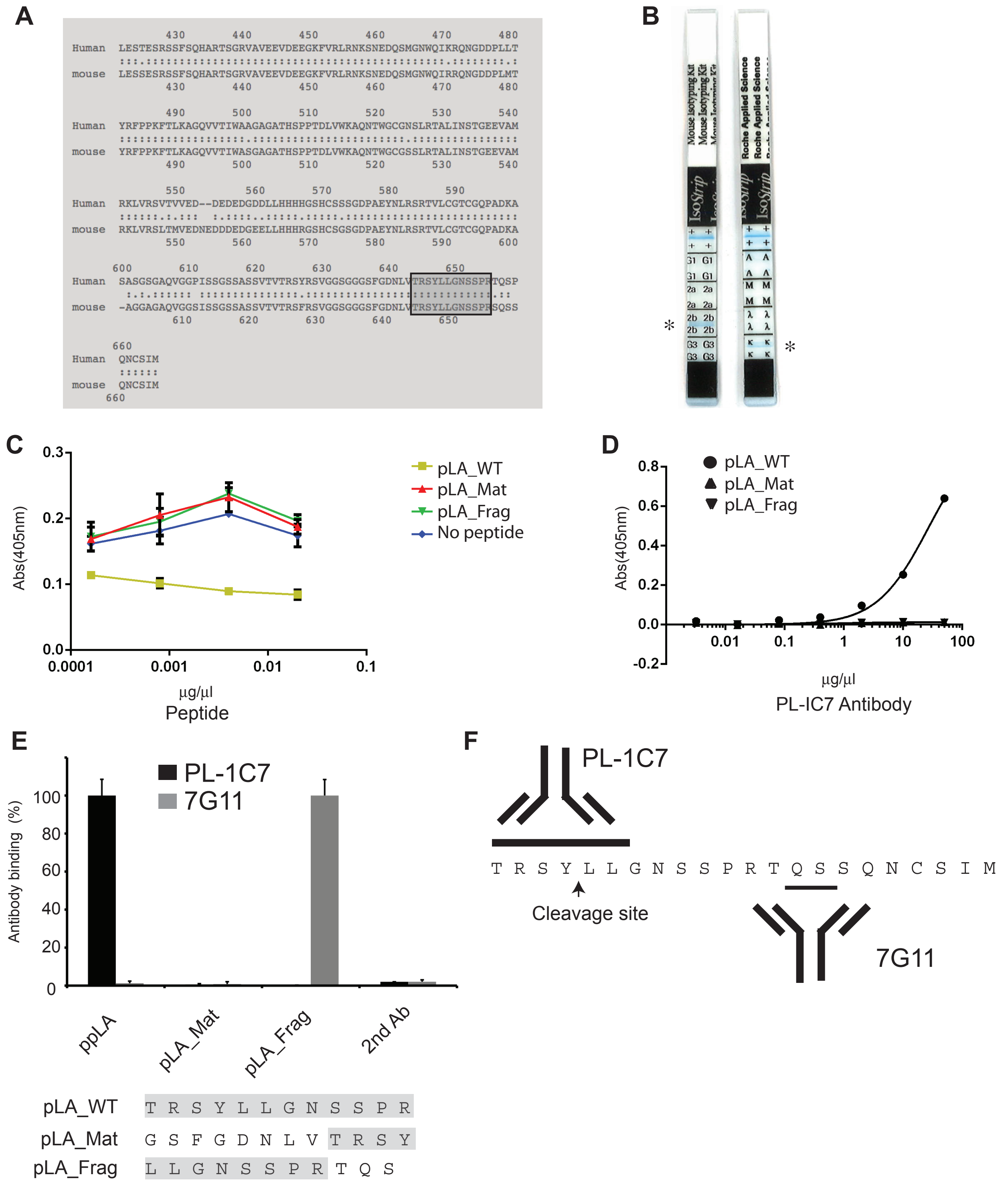

Figure S1. PL-1C7 antibody specifically recognizes prelamin A but not mature lamin A peptides after ZMPSTE24 cleavage. **A.** Sequence analysis of the carboxyl terminus of mouse and human prelamin A confirms 100% of identity for the peptide sequence used for the development of PL-1C7 (Box). **B.** Characterization of PL-1C7 Isotype. Isotype strips show that PL-1C7 is IgG2b kappa. **C.** Peptides containing ZMPSTE24-generated lamin A fragments do not compete against the intact TRSYLL sequence for PL-1C7 binding. PL-1C7 antibody was pre-incubated with pLA_WT, pLA_Mat, pLA_Frag or without peptide, and allow to bind to ELISA plates coated with pLA_WT peptide (wild type sequence). **D.** PL-1C7 shows high specificity for the pLA_WT sequence. Increasing concentrations of PL-1C7 antibody were incubated onto ELISA plates coated with pLA_WT, pLA_Mat, pLA_Frag. **E.** PL-1C7 binding does not overlap with the epitope of the rat anti-prelamin A 7G11 antibody. 7G11 antibody potentially targets the TQS epitope. **F.** Diagram depicts the potential target sequences for both PL-1C7 and 7G11 antibodies.

Figure S1. Casasola et al. 2016

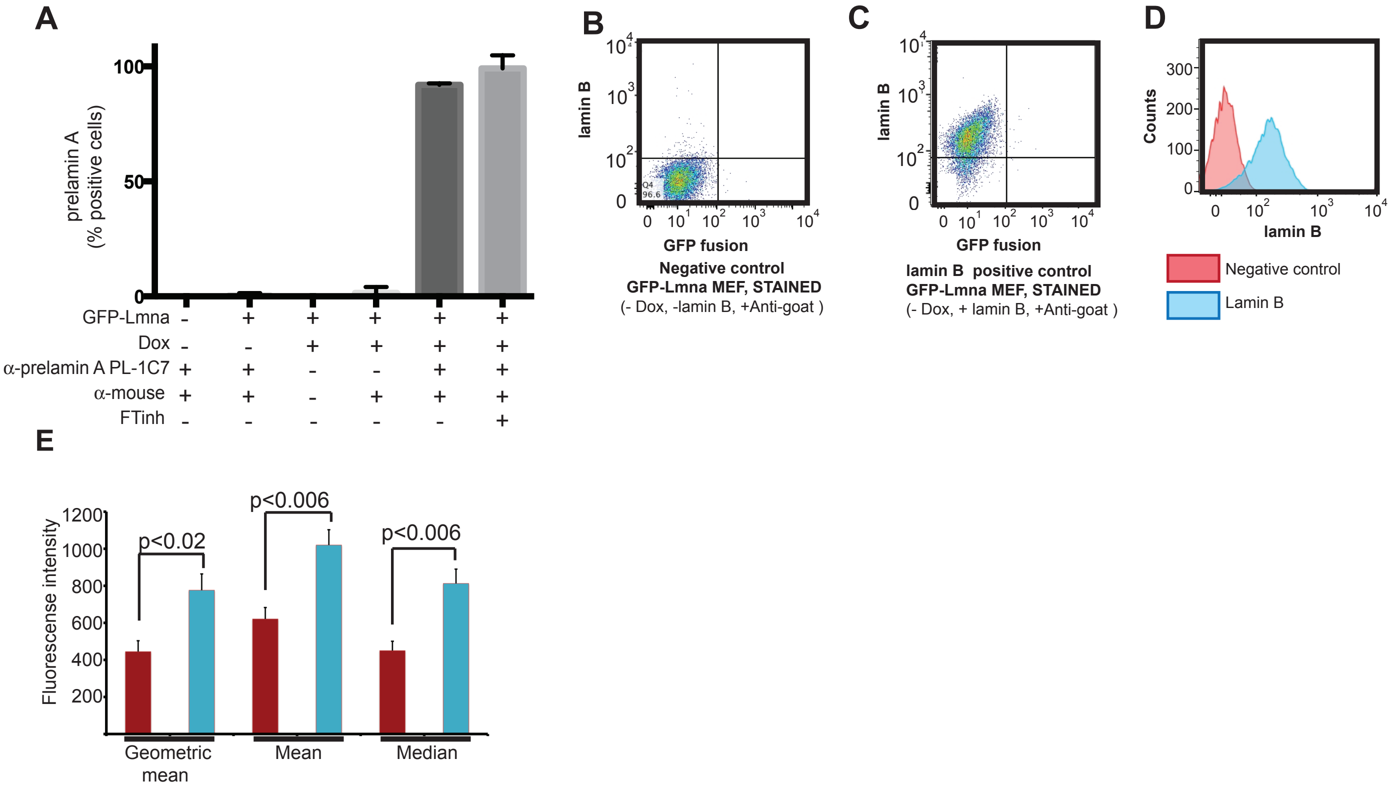

Figure S2. Prelamin A quantification by intracellular flow cytometry. **A.** Intracellular flow cytometry validation using PL-1C7 after Dox and Lonafarnib treatment. FTinh increases prelamin A accumulation but not the percentage of positive cells. **B and C.** IFC validation using lamin B detection with no primary antibody (B) or anti-lamin B antibody (C). Cells were not treated with Dox. **D.** Histogram of lamin B detection in *Lmna-/-* MEFs. **E.** IFC analysis of GFP-*Lmna* MEFs upon FTinh treatment using PL-1C7 antibody. Mean, geometric mean and median are shown.

Figure S3. Casasola et al. 2016

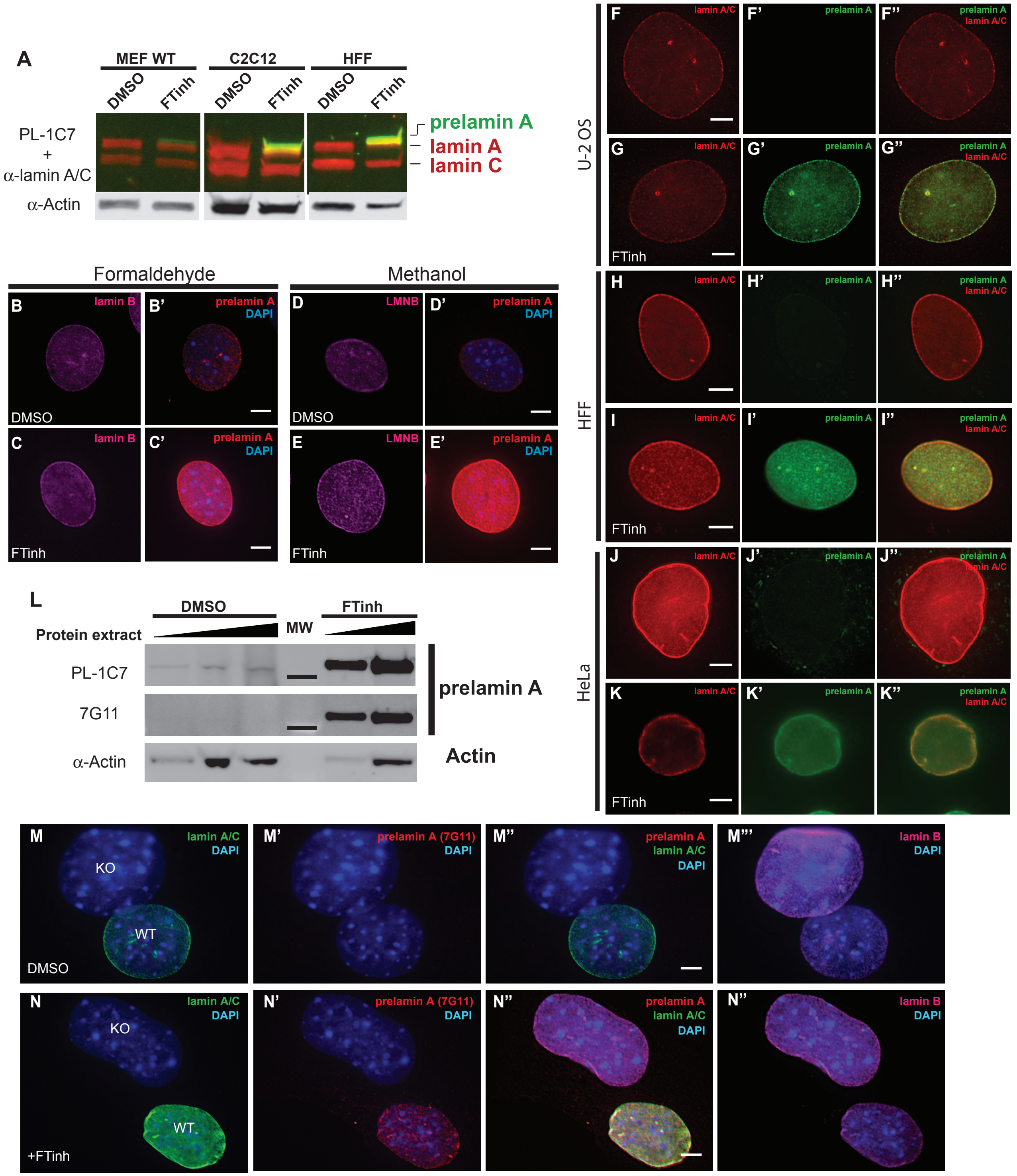

Figure S3. Prelamin A accumulation and localization analysis in cell lines treated with FTinh. **A.** Dual immunoblotting of protein extracts from wildtype MEFs, C2C12 and human forskin fibroblasts treated with FTinh. Extracts were transferred to a membrane and incubated with PL-1C7 (prelamin A; 800 nm channel) and anti-lamin A/C (700 nm channel) antibody. Actin was used as a loading control. **B-E.** To establish that the diffuse prelamin A accumulation upon FTinh treatment detected by PL-1C7 does not depend on the fixation method, C2C12 cells treated with DMSO (B and D) or FTinh (C and E) were fixed side-by-side with paraformaldehyde (B and C) or methanol (C and D). Cells were stained with anti-prelamin A PL-1C7 antibody and counterstained with DAPI. Lamin B was used as control. **F and G.** Control or FTinh treated U-2 OS cells were immunostained with PL-1C7 (prelamin A; green) and anti-lamin A/C (red) antibodies. **H and I.** HeLa were treated and stained as in A and B. **J and K.** Human Foreskin fibroblast (HFF) were treated and stained as in A and B. **L.** PL-1C7 has higher sensitivity to identify prelamin A than 7G11 antibody. Increasing amounts of protein extracts from C2C12 cells with and without FTinh treatment were resolved by PAGE and transferred to membranes. Membranes were probed with anti-prelamin PL-1C7 and 7G11 antibodies and binding evaluated by chemiluminescence. b-actin was used as loading control. Molecular weight (MW; 70KDa) band is shown. **M and N.** Co-cultured *Lmna-/-* (KO) and wild type (WT) MEFs with or without FTinh treatment were stained with anti-prelamin A 7G11, anti-lamin A/C, and anti-lamin B antibodies and counter stained with DAPI. Anti-lamin A (LMNA) antibody was used to distinguish between KO and WT cells. Scale bar= 5 μm.

Figure S4. Casasola et al. 2016

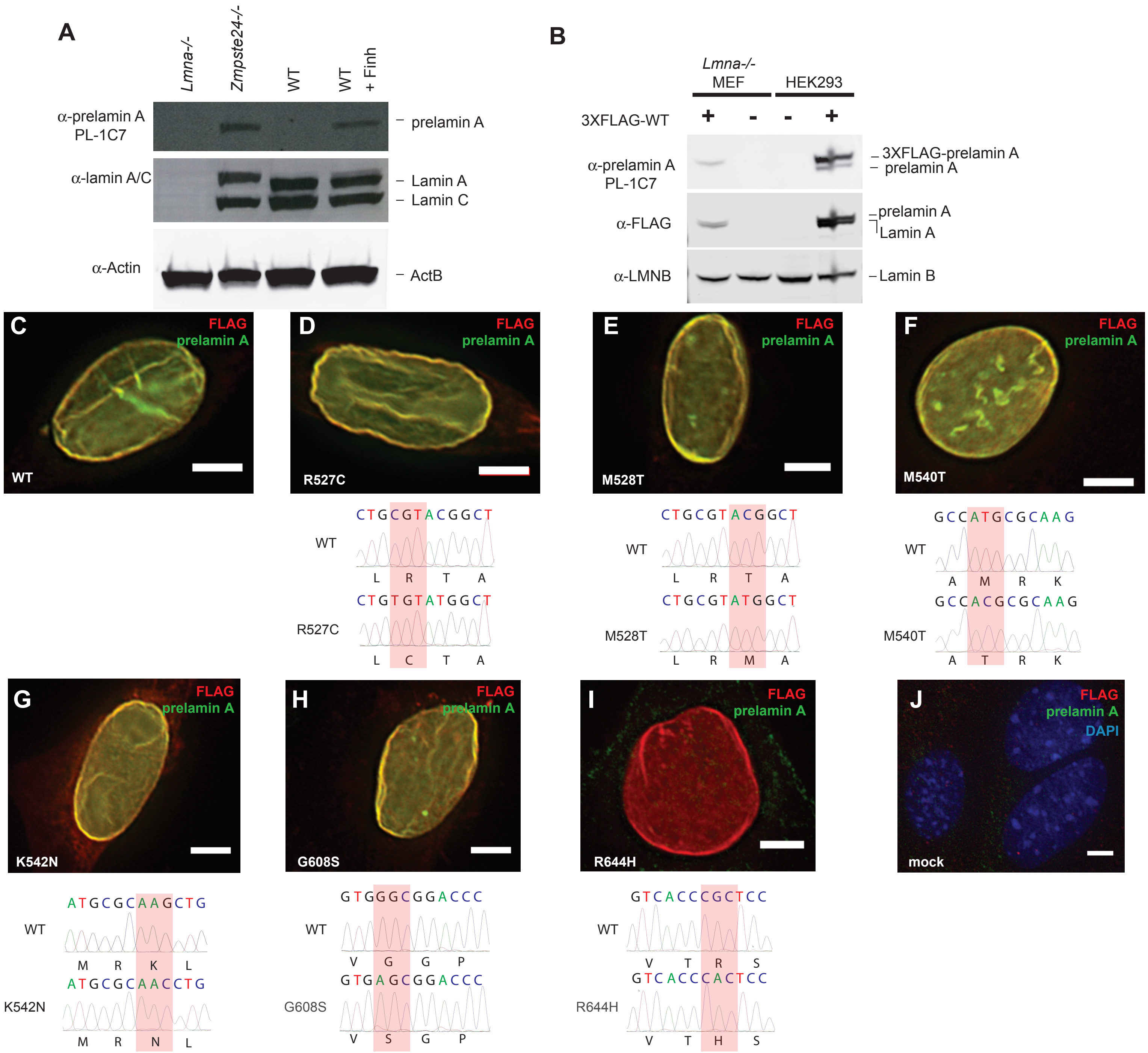

Figure S4. Alterations in the prelamin A processing pathway and laminopathy-associated mutations differ in their effect on prelamin A accumulation. **A.** Lack of ZMPSTE24 expression or activity results in prelamin A accumulation. Protein extracts from Zmpste24-/-, Lmna-/-, FTinh-treated and WT MEFs were blotted with anti-prelamin A PL-1C7 antibody. Anti-lamin A/C and anti-β actin antibodies were used as controls. **B.** Transient transgene expression does not generate saturating levels of prelamin A. Lmna-/- MEF and HEK293 cells were transfected in parallel with wild type 3XFLAG LMNA encoding plasmid and incubated for 48 h. Western blotting showed that the tagged proteins are expressed in lower level in Lmna-/- MEF cells than in HEK293 cells. Importantly, we detect similar levels of the tagged prelamin A levels in Lmna-/- MEF cells to those detected in HEK293 cells with endogenous prelamin A. Additional immunoblotting was performed with anti-FLAG antibody, which detects both the mature 3XFLAG-lamin A and 3XFLAG-prelamin A. Lamin B was used as loading control. **C-I.** 3XFLAG fusion proteins localize at the nuclear periphery. Transfected cells were stained with anti-prelamin A PL-1C7 and anti-FLAG antibody to establish proper localization of the tagged proteins to the nuclear envelope only. Laminopathy-associated LMNA variants R527C (D), T528M (E), M540T (F), K542N (G), G608S (H) and R644H (I) localize at the nuclear envelope. **J.** mock transfected *Lmna-/-* MEF cells were stained in parallel as in C-J. Chromatograms for each mutation are shown. Scale bar= 5 μm.

Table S1. Lamin A point mutations associated with laminopathies

Aminoacid	Protein nomenclature	cDNA Nomenclature	Mutational event	Disorder	References
527	R527C	c.1579C>T	C->T	Progeria	Cao and Hegele, 2003; Liang, etal. 2009.
528	T528M	c.1583C>T	C->T	Progeria	Verstraeten, et al. 2006; Savage, 2004.
540	M540T	c.1619T>C	T->C	Progeria	Verstraeten, et al. 2006; Plasilova, 2004.
542	K542N	c.1626G>C	G->C	Progeria	Plasilova, 2004
608	G608S	c.1822G>A	G->A	Progeria	Eriksson, *et al.* 2003; Cao and Hegele, 2003.
644	R644H	c.1931G>A	C->T	L-CMD	Genschel, et al. 2001; Mercuri. 2009; Csoka, 2004; Muntoni, et al, 2006; Rankin, et al. 2008; Pasotti, et al, 2008; Moller, 2009; Scharner, 2011; De vos, et al 2011
